# The global geography of plant invasion risk under future climate and land-use changes

**DOI:** 10.1038/s41559-026-03040-2

**Published:** 2026-03-27

**Authors:** Ali Omer, Stefan Dullinger, Johannes Wessely, Bernd Lenzner, Adrián García-Rodríguez, Anna Schertler, Dietmar Moser, Andreas Gattringer, Amy J. S. Davis, Wayne Dawson, Trevor S. Fristoe, Matthias Grenié, Nicole L. Kinlock, Holger Kreft, Jan Pergl, Petr Pyšek, Mark van Kleunen, Patrick Weigelt, Marten Winter, Damaris Zurell, Franz Essl

**Affiliations:** 1 Division of BioInvasions, Global Change & Macroecology, Department of Botany and Biodiversity Research, University of Vienna, Vienna, Austria; 2 Department of Forest Management, Faculty of Forestry, University of Khartoum, North Khartoum, Sudan; 3 Division of Biodiversity Dynamics & Conservation, Department of Botany and Biodiversity Research, University of Vienna, Vienna, Austria; 4 Ecology, Department of Biology, University of Konstanz, Konstanz, Germany; 5 Department of Evolution, Ecology and Behaviour, Institute of Infection, Veterinary and Ecological Sciences, University of Liverpool, Liverpool, UK; 6 Department of Biology, University of Puerto Rico - Río Piedras, San Juan, Puerto Rico; 7 University Grenoble Alpes, University Savoie Mont Blanc, CNRS, LECA, Grenoble, France; 8 Biodiversity, Macroecology & Biogeography, University of Göttingen, Göttingen, Germany; 9 Centre of Biodiversity and Sustainable Land Use (CBL), University of Göttingen, Göttingen, Germany; 10 Campus Institute Data Science (CIDAS), University of Göettingen, Göttingen, Germany; 11 Department of Invasion Ecology, Institute of Botany, Czech Academy of Sciences, Průhonice, Czech Republic; 12 Department of Ecology, Faculty of Science, Charles University, Prague, Czech Republic; 13 Zhejiang Key Laboratory for Restoration of Damaged Coastal Ecosystems & Zhejiang Provincial Key Laboratory of Plant Evolutionary Ecology and Conservation, Taizhou University, Taizhou, China; 14 Department of Environmental Science, Radboud Institute for Biological and Environmental Sciences (RIBES), Radboud University, Nijmegen, The Netherlands; 15 German Centre for Integrative Biodiversity Research Halle-Jena-Leipzig – iDiv e. V., Leipzig, Germany; 16 Institute for Biochemistry and Biology, University of Potsdam, Potsdam, Germany

**Keywords:** Invasive species, Ecological modelling

## Abstract

Biological invasions by plants pose a growing threat to biodiversity. Here we model potential current and future distributions of 9,701 naturalized alien plant species to project their potential spread by the end of the twenty-first century. Our analysis reveals that 33.9% of the global land surface is suitable for at least 10% of these species, identifying key hotspots for invasion. Under future climate and land-use scenarios, these hotspots are projected to expand moderately to 37.7% and 36.6% of land surface under mild and severe changes, respectively. However, this moderate absolute increase conceals substantial spatial shifts in hotspot locations, with expansion into currently cooler regions and contraction in hotter, drier areas. Additionally, we observe substantial species turnover within regional naturalized plant pools, indicating not only increases in plant invasion risk, but also shifts in the composition of the alien plant species pools. Our models predict regionally divergent responses of naturalized plant richness and species pool composition to climate and land-use changes.

## Abstract (web summary)

The spread of non-native species is likely to be compounded by further climate change and land-use intensification. Here the authors assess the potential distribution of 9,701 naturalized non-native plant species at a 10 × 10-km grid resolution and project shifts under two Shared Socioeconomic Pathways.

## Main

The deliberate and unintentional translocation of species worldwide has accelerated considerably in recent decades, resulting in a massive global redistribution of plant species^1,2^. As the drivers of biological invasions continue to intensify, the spread and naturalization of alien plants are anticipated to continue in the next decades^3–5^. Currently, at least 16,429 vascular plant species are known to have naturalized, that is, they have successfully established self-sustaining populations in at least one region outside their native range^6^. Naturalized alien plants have caused the loss of floristic uniqueness in various parts of the world through the taxonomic, functional and phylogenetic homogenization of the global flora^7–9^. A subset of naturalized alien plants (approximately 6% (ref. ^3^)) are causing negative environmental impacts and massive socioeconomic costs (these plant species are referred to as invasive alien plants)^10–12^. Accordingly, biological invasions are a major threat to biodiversity and human livelihoods^3^.

During the past decade, the first comprehensive resource on the regional distribution of the world’s naturalized alien flora has been compiled (that is, the Global Naturalized Alien Flora database, GloNAF^13^). While numerous studies have leveraged GloNAF to address macroecological questions on the extent and mechanisms of plant invasions^14–19^, large knowledge gaps persist, in particular with respect to how the distribution of the naturalized flora will respond to environmental change at finer spatial grains^5,20^. Assessments of future naturalized alien plant distributions have so far used coarse spatial resolutions^21^, focused on well-studied regions^22–25^, or selected subsets of species^26,27^, for example, 100 of the worst invaders^28^. Yet, a comprehensive assessment of how ongoing and future climate and land-use changes may alter the potential distributions of the global naturalized alien flora and grid-cell level naturalized species composition is lacking. Addressing this research gap is critical for understanding potential naturalized alien plant species redistribution under climate and land-use changes, and for informing effective and proactive biosecurity, conservation and policies by, for example, using spatially refined (beyond regional checklists) species distribution knowledge.

Here we provide projections of potential current and future distributions of the naturalized alien plant species pool by the end of the twenty-first century under contrasting scenarios of climate and land-use changes. Our analysis encompasses 9,701 plant species, representing 70.2% of all plant species worldwide that have naturalized in at least one region of the world^6^ and have sufficient occurrence information to enable modelling. We applied ensemble species distribution models (SDMs) with a spatial resolution of 10 × 10-km grid cells, considering climate, land use and soil pH as predictor variables. This approach enables us to do the following: (1) project current and future potential distributions of the global naturalized alien flora; (2) map potential hotspots of plant invasions; (3) predict shifts in these hotspots across biogeographic biomes; and (4) assess the potential turnover within regional naturalized alien plant species pools.

## Results and discussion

### Current potential distribution of naturalized flora

**Fig. 1:**
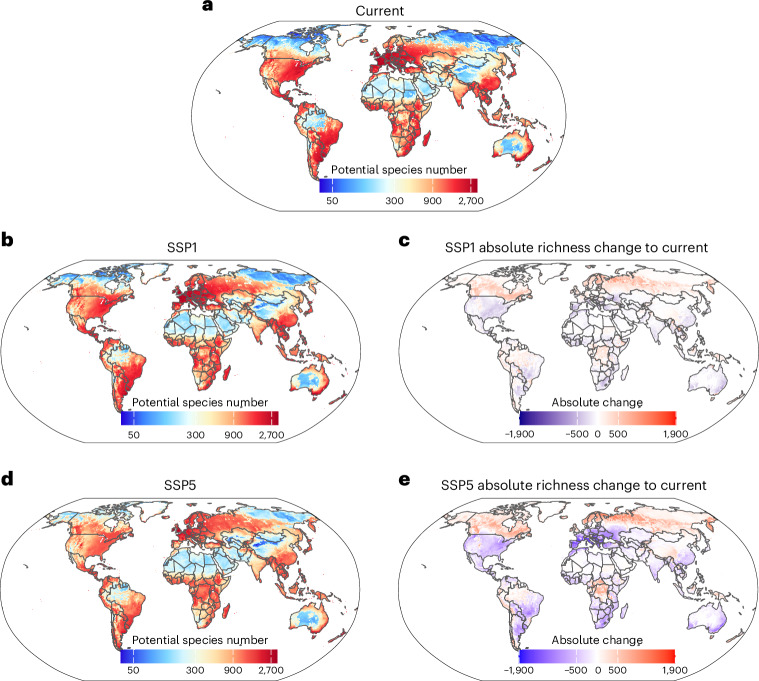
Predicted global patterns of naturalized alien plant species richness across 10 × 10-km grid cells. **a**,**b**,**d**, Maps of naturalized alien plant species richness under current environmental conditions (**a**), and future mild (**b**) and severe (**d**) climate and land-use change scenarios of projected socioeconomic global changes until the end of the twenty-first century (2071–2100). Predicted richness is based on the ensemble of four models using two different environmental predictor sets and two GCMs (single GCM maps are provided in Supplementary Fig. 1). **c**,**e**, Absolute change in relation to current richness under future change scenarios SSP1 (**c**) and SSP5 (**e**). For proportional changes in relation to current species richness under future change scenarios see Supplementary Fig. 2. Colour coding of species numbers is on a log_10_ scale and all maps use Robinson projection. Unscaled maps of potential current and future distributions are provided in Supplementary Fig. 3.

Our models showed strong spatial variation in potential distributions of the naturalized alien plant species under current environmental conditions. Some regions are predicted to provide suitable conditions for up to 3,443 (out of 9,701 considered) naturalized plant species per 10 × 10-km grid cell, while others may support as few as 23 plant species (median (interquartile range): 537 (192–1,250) species; Fig. 1a). Centres of potential richness of naturalized alien plant species are located in temperate regions of the northern (that is, Europe, Northern America) and the southern (that is, parts of South America, Australia, New Zealand) hemispheres (Fig. 1a). This aligns with the current observed global pattern of naturalized alien plant species^29^. The predicted pattern of naturalized alien plant species richness (Fig. 1a) correlates positively with the human footprint index^30,31^ and negatively with the ecosystem integrity index^32^. This suggests that anthropogenic activities increase global invasion risks. Regions with high human activity accordingly have high levels of anthropogenic disturbances, removing native plants and providing establishment opportunities for alien plants, and also experience increased propagule pressure of alien plants due to the movement of goods, people and resources^33^.

Notably, our models also predict high potential naturalized alien plant richness in (sub)tropical regions of South America, sub-Saharan Africa and Southeast Asia, contrasting with current observations^29^. The discrepancy between observed and predicted numbers of naturalized alien plant species in these (sub)tropical regions might be due to undersampling^34,35^, dispersal limitation between globally distant tropical regions^36^, high invasion resistance from the diverse native flora^14,37^, lower historic propagule pressure^38,39^ or the combination of all these factors. Species native to (sub)tropical regions were over-represented among the unmodelled species (Supplementary Fig. 4). This indicates that our results are probably conservative and may thus underestimate the potential invasion risk in these regions. To assess the potential influence of unmodelled species on our results, we added the number of unmodelled species to potential naturalized alien plant species richness in grid cells within naturalized regions according to GloNAF. Although this increased the potential invasion risk, particularly in (sub)tropical regions, the overall pattern remained unchanged (Supplementary Fig. 5). Thus, although our results support previous studies documenting disproportionately high numbers of naturalized alien plant species in temperate regions^1,2,29^, we additionally identify a high potential invasion risk within (sub)tropical environments (Fig. 1a).

**Fig. 2:**
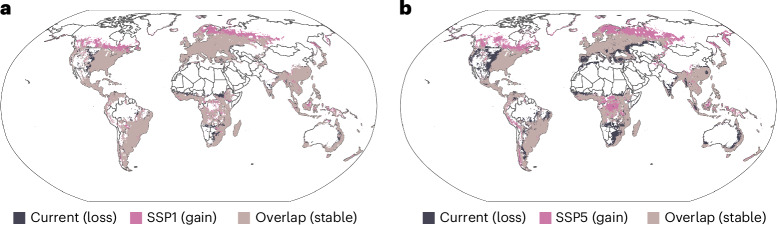
Global hotspots of plant invasion risk. **a**,**b**, Change in proportion and spatial distribution of plant invasion hotspots under current environmental conditions and mild (**a**) and severe (**b**) environmental change scenarios until the end of the twenty-first century (2071–2100). Invasion hotspots were defined as grid cells that are predicted to be suitable to >10%, (that is, 970) of the modelled species.

**Fig. 3:**
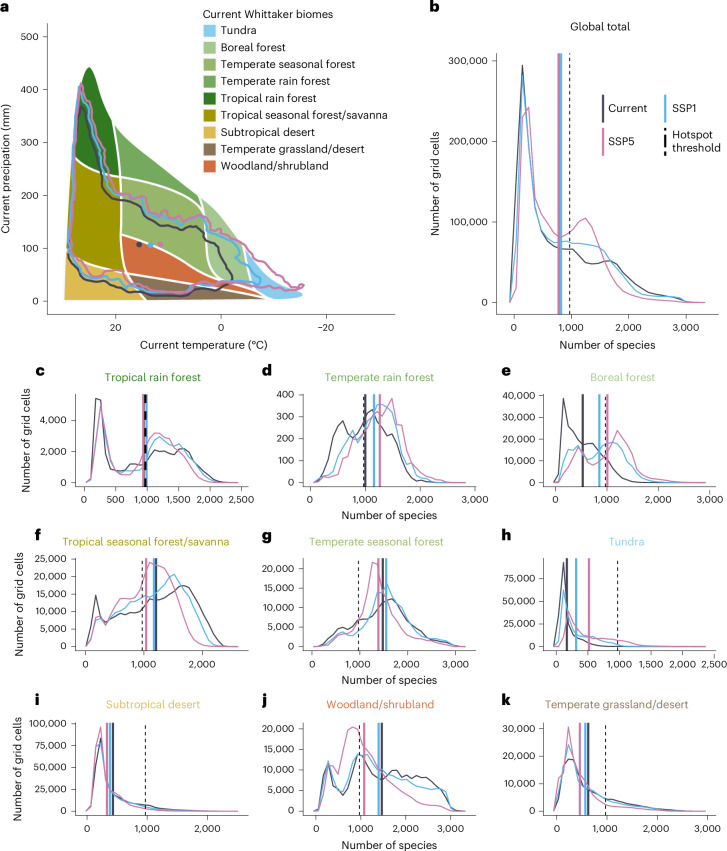
Global current and future potential plant invasion risk across current biomes. **a**, Contour lines show the 95% kernel density and centroids (coloured dots) of the projected richness of naturalized alien plant species under current (dark grey), and mild (blue) and severe (pink) climate and land-use change scenarios until the end of the twenty-first century (2071–2100). **b**–**k**, The density plots show the distribution of projected alien plant richness per grid cell in all biomes (**b**) and in each of the biomes separately (**c**–**k**). Solid vertical lines in each density plot represent the mean plant richness under the current conditions and under future climate scenarios. Dashed vertical lines represent the hotspot threshold, that is, 970 species.

To identify potential hotspots of plant invasions, we applied a cut-off threshold of suitability for 10% of the modelled species pool under current environmental conditions (that is, cells with suitable conditions for >970 species were considered hotspots^22^). Under current environmental conditions, potential hotspots are projected to cover a total of 33.9% of the global landmass (Fig. 2). To test whether potential invasion hotspots occur in regions with high native plant diversity, we correlated native plant species richness^40^ with the potential naturalized alien plant species richness and found a significant positive association (Supplementary Fig. 6). Thus, our results indicate that centres of native plant richness will potentially accumulate more naturalized plant species, supporting the ‘rich get richer hypothesis’ on the landscape scale^41^. The distribution of invasion hotspots differed substantially across biomes (Fig. 3), with woodlands, shrublands and temperate or tropical seasonal forests that occur across temperate to subtropical climates (see Whittaker plot in Fig. 3a) having considerably higher potential naturalized plant richness than the global average. Conversely, tundra, boreal forest, desert and grassland biomes have lower projected current richness of naturalized alien plant species per grid cell (Fig. 3).

### Future potential distribution of naturalized flora

Under the mild climate and land-use change scenario, potential alien naturalized plant species richness showed inconsistent patterns of losses and gains, with some regions experiencing increases while others showed decline. Overall, potential alien naturalized plant species richness across all 10 × 10-km grid cells is projected to increase to a median of 662 naturalized alien plants (interquartile range: 233–1,294), while under the severe climate and land-use change scenario it is modelled to increase to a median of 669 species (interquartile range: 268–1,196). Although the potential naturalized alien plant species richness is projected to increase across the globe, there is spatial variability in losses and gains depending on how the climate will change in the future (Fig. 1c,e). The analysis of invasion hotspots yielded similar results. In the future, the proportion of global hotspots is predicted to increase to 37.7% and 36.6% under mild and severe climate and land-use change scenarios, respectively, compared with the current 33.9% (Fig. 2).

**Fig. 4:**
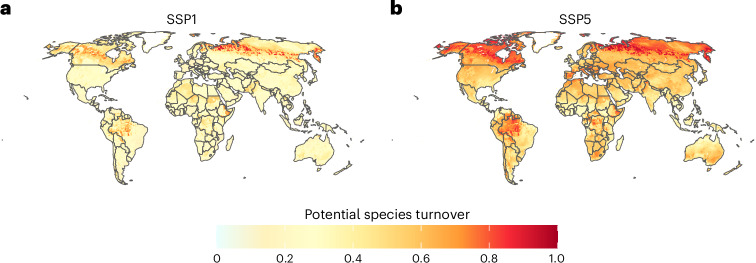
Projected turnover of naturalized alien plant composition per 10 × 10-km cell. **a**,**b**, Turnover index values vary between 0 (no change in suitable species list) and 1 (cell has not even one suitable species in common under current and future conditions). Compared are current conditions with mild (**a**) and severe (**b**) environmental change scenarios until the end of the twenty-first century.

Our results also indicate considerable spatial and compositional re-arrangement of potential naturalized alien plant richness and invasion hotspots (Figs. 2 and 3). Under climate and land-use change until the end of the twenty-first century, the centroid and 95% contour line of naturalized alien plant richness per grid cell are projected to expand polewards into currently cold regions (Fig. 3a). As expected, this shift is more pronounced under the severe Shared Socioeconomic Pathway 5 (SSP5) scenario than under the mild SSP1 scenario. Moreover, there are divergent patterns for individual biomes. Under the SSP5 scenario, substantial increases in alien plant richness are projected for cold regions compared with current averages, while losses predominate in precipitation-limited environments (that is, grassland, shrubland, desert and savanna biomes; Fig. 3c–k). Under the SSP1 scenario, the patterns are qualitatively similar, but somewhat less pronounced. These spatial shifts in naturalized alien plant species richness are also accompanied by substantial turnover in species pool composition (Fig. 4). Temperate regions are projected to experience high turnover rates, particularly under the extreme SSP5 scenario, with some grid cells reaching nearly complete turnover of their suitable species pool (index values close to 1 in Fig. 4). Turnover in temperate regions is mostly driven by the increases and decreases of potential naturalized alien plant species richness (Supplementary Figs. 7 and 8). In contrast, high species turnover is also modelled in tropical regions, but the underlying dynamics appear to differ. As the number of naturalized plant species per grid cell is relatively stable over time in the tropics (Fig. 1), it is apparently species replacement rather than net changes in naturalized alien plant richness that drives turnover.

The invasion hotspot shifts correspond to the redistribution of potential naturalized plant species richness patterns. While under the SSP1 change scenario, 93.4% of current hotspots are projected to remain hotspots until the end of the twenty-first century (Fig. 2a), and this percentage shrinks to only 82.1% under the SSP5 scenario (Fig. 2b). In particular, many new invasion hotspots are projected to arise in currently cold boreal regions of the Northern Hemisphere, while many current hotspots will be lost in currently temperate and subtropical semi-arid regions. The predicted emergence of novel regional naturalized plant species pools induced by severe climate changes also indicates that these new invasion hotspots might functionally differ from current ones due to altered species composition. The predicted poleward shift of invasion hotspots is consistent with recent regional assessments^42^, and corresponds to the predominant impact of temperature on the potential niches of naturalized plant species in our models. Specifically, annual mean temperature (bio1), temperature annual range (bio7), temperature seasonality (bio4) and mean temperature of the warmest quarter (bio10) show the highest variable importance in our models, whereas precipitation metrics (bio12, bio15, bio18), land use and soil pH contribute less overall (Supplementary Fig. 9). The predominantly temperature-driven shift in invasion risk centroids reinforces this pattern (Fig. 3a). As a corollary, current cold boreal and polar regions, which now have relatively low numbers of naturalized plant species, may become highly susceptible to plant invasions in the future, adding further pressure on these particularly sensitive ecosystems^43–45^ and their species-poor but distinctive flora^40^.

### Projection uncertainties

Predicting species distributions is challenging due to multiple uncertainties. In our study, we minimized uncertainties associated with SDM techniques, predictors and future variability. To ensure accuracy, we performed an exploratory analysis with a subset of species to identify the most appropriate set of pseudo-absences (Supplementary Methods 1). Additionally, we aggregated the different SDMs into a single consensus model to produce robust projections of invasion risks. Nevertheless, uncertainties remain and in future projections soil pH and biome location are assumed unchanged, even though they may shift under future climates. Projection uncertainties (measured as the coefficient of variation) were highest in regions with extreme climatic conditions and low accessibility (Supplementary Fig. 10), such as around the Sahara Desert and in subarctic regions. This probably stems from lower accuracy in species distribution data and environmental data availability in these areas^34,46^. As expected, uncertainty was higher under the severe (Supplementary Fig. 10b) compared with the mild change scenario (Supplementary Fig. 10a). Regions with high uncertainty in our projections might also show novel environmental conditions under future scenarios, which could potentially lead to an over- or underestimation of the effect of future climate on invasion risk^47,48^.

Additional factors such as geographical barriers, differences in propagule pressure and lag times in spread after introduction might also bias our projections. We account for that by recalculating naturalized alien plant richness and invasion hotspots exclusively based on predicted suitable grid cells located on continents where a given species is already reported to be naturalized. The results of this analysis showed a lower overall invasion risk compared with the initial non-filtered projections (Supplementary Figs. 11–13). The reduction in predicted invasion risk was more pronounced in the Americas (South and North America) compared with other continents, particularly Europe, where human activities have probably facilitated plant dispersal more and over longer historical periods. Importantly, this sensitivity analysis did not change our main conclusion. Thus, although our projections of potential current and future distributions of naturalized plants need careful interpretation in regions with high uncertainty, they offer valuable resources for biological invasions management, conservation prioritization and biodiversity monitoring.

The increase in global accumulation of alien plant species is expected to continue without signs of saturation^49^. Consequently, the potential distribution of global naturalized plant species richness we present here is likely to be surpassed due to the introduction of new species. To account for that, we performed a sensitivity analysis using the Alien Species First Records dataset^50^. Specifically, we assessed the potential naturalized alien plant species richness in grid cells by using subsets of alien species with first records up to certain time points (that is, 1850, 1900, 1950). This allowed us to examine whether patterns of naturalized species richness change as new species are added to the naturalized plant species pool. We found that the overall pattern remained unchanged, meaning that alien plant species are being integrated into the global naturalized species pool with comparable environmental tolerances in similar regions over time (Supplementary Fig. 14).

Beyond these quantified sources of uncertainty, our models do not encompass all possible drivers of plant invasion risk. In particular, future plant invasion risks are likely to be shaped by complex socioeconomic activities such as plant introduction bias and ongoing emergence of new alien plant pools associated with changing global trade patterns. To address these limitations, future research should integrate environmental suitability with species introduction history and socio‑economic indicators (for example, trade intensity, transport networks, urbanization and biosecurity capacity). In addition, future studies should adopt dynamic species pool scenarios that reflect evolving global trade and horticultural demand.

## Conclusion

Our global projections of naturalized alien flora suggest that naturalized alien plant species richness across the globe, as well as the proportion of regions classified as invasion hotspots, will show divergent regional trajectories. Future climate and land use are projected to have a massive impact on which species find suitable conditions where, and on the location of potential plant invasion hotspots. With rising temperatures, climate change is likely to increase invasion risk in current boreal and polar regions, and lead to decreases in increasingly dry temperate and subtropical semi-arid regions. These contrasting regional trends reveal complex interactions of environmental change and plant invasions, and highlight the need for tailored proactive management strategies that account for both current conditions and the specific nature of predicted climate and land-use changes.

## Methods

### Species selection and occurrence data

We based the selection of the global naturalized alien plant species pool on the most comprehensive data source, that is, the GloNAF database. We used GloNAF v.2.0^6^, which contains 16,429 taxa (that is, species, subspecies and varieties) that are naturalized in at least one region globally. Infraspecific taxa (for example, subspecies, varieties) were removed. As a result, the final list included 13,810 naturalized alien plants with accepted binomial names.

Then, we retrieved all geo-referenced occurrences (both from the native and alien ranges to capture the broadest possible of the realized niche) from the Global Biodiversity Information Facility (GBIF; https://www.GBIF.org, accessed 15 February 2024, 10.15468/dl.3hmh4k). A total of 13,724 species were found in GBIF (exact match), resulting in a total of 161,715,874 geo-referenced occurrence records. Following standard practices^51^, we checked and cleaned these occurrence records. Erroneous records (that is, those assigned to ocean surfaces, administrative capitals or headquarters of GBIF, and those lacking one or both coordinates) were automatically removed using the CoordinateCleaner package in R^52^. Additionally, we removed duplicate data points (that is, multiple occurrence records within each 10 × 10-km grid cell) to avoid pseudoreplication and reduce sampling bias. Furthermore, we environmentally filtered occurrence data to account for potential sampling biases, as species might be disproportionately sampled from specific environmental regions (for example, areas with favourable climates) and to ensure a balanced representation of species occurrences across different environmental conditions. To this end, we filtered species occurrences based on an environmental filtering approach proposed by ref. ^53^ and implemented in the R package flexsdm using the occfilt_env() function^54^. Specifically, we constructed a regular multidimensional grid over the environmental space using the climatic variables mentioned below, using six bins to define cell size. Then, a single occurrence point was randomly selected within each grid cell of the multidimensional environmental space. Finally, to ensure accurate and robust SDM predictions, we only kept species with at least 30 occurrence records^55^. The final dataset comprised 9,701 species, that is, 70.2% of all 13,810 global naturalized alien plants.

### Environmental variables

We initially aimed to consider a wide range of environmental predictors related to climate, physico-chemical soil properties, and land use and cover^56^. However, as several of these predictors are highly correlated and hence should not be used in the same model^57^, we calibrated our models with two alternative sets of environmental predictors that are known to affect plant distribution. The first set of environmental variables includes: (1) annual mean temperature (bio1); (2) temperature annual range (bio7): (3) annual precipitation (bio12); (4) precipitation seasonality (coefficient of variation; bio15); (5) land use; and (6) soil pH. The second set of environmental variables includes: (1) temperature seasonality (standard deviation; bio4); (2) mean temperature of warmest quarter (bio10); (3) precipitation seasonality (coefficient of variation; bio15); (4) mean monthly precipitation amount of the warmest quarter (bio18); (5) land use; and (6) soil pH. We extracted climatic variables (average of the baseline period 1979–2013) at a 30-arcsec resolution (~1 km) from CHELSA v2.1^58^. Moreover, we extracted data on topsoil pH (that is, the first 15 cm of soil) at a 1-km resolution from the global gridded soil information database SoilGrids^59^. Finally, for land use and land cover, we used the proportion of primary and secondary land cover (that is, land with natural vegetation that has not been subject to human activity since 1500 CE). Land use data (average of the baseline period 1979–2013) are available at a ~25-km resolution from the Land-Use Harmonization 2 dataset (LUH2)^60^. All environmental predictors were harmonized to a resolution of 10 × 10 km. Using pairwise Pearson correlation coefficients, we checked that no pairs of predictors exceeded correlations of |*r*| = 0.70 to avoid unreliable results due to collinearity^57^ (Supplementary Fig. 15).

For projecting potential future distributions of naturalized alien plants until 2100, we used the SSP framework for the period 2071–2100. We used a moderate (SSP1—Sustainability: Taking the Green Road) and a severe (SSP5—Fossil-fuelled Development: Taking the Highway) environmental change scenario; the corresponding future climate and land-use data were also extracted from CHELSA and LUH2 mentioned above. For soil pH, owing to lack of global scenarios, we assume in our future projections no change in soil pH between current and future conditions. The substantial differences in future climate data introduced by different general circulation models (GCMs) potentially result in differences in future species distribution projections^61^. To account for that, we used a set of five GCMs and chose the two most different ones (that is, ukesm1-0-ll and mpi-esm1-2-hr) in terms of predicted number of species per grid cell to account for uncertainties related to GCMs within a given climate change scenario. To reduce computational effort, the selection of these SSPs and GCMs was based on a subset of 100 randomly selected species (Supplementary Methods 1, and Supplementary Figs. 16 and 17).

### Species distribution modelling

To model the potential current and future distributions of the global naturalized alien plants, we employed ensemble SDMs using the biomod2 R package version 4.2-5-2 (ref. ^62^). We used four modelling algorithms: two regression techniques (generalized linear models; general additive models) and two machine learning techniques (random forests,; boosted regression trees). We maintained the default settings of these four modelling algorithms as given in biomod2. As we only have presence data, we had to generate pseudo-absence (that is, background) data. We used 100 randomly selected species to identify the most appropriate approach of selecting the number and distribution of pseudo-absences (Supplementary Figs. 18 and 19). Based on that, we generated as many pseudo-absence records for each species as we had presence records. For each species, pseudo-absence records were drawn outside a suitable area estimated by a surface range envelope model from the presence records of that species. To account for sampling bias in the presence data, we applied the target-group approach^63^ that corrects for variation in sampling intensity. We used the sampling effort index developed by ref. ^34^, thus, we selected more pseudo-absences from areas with higher sampling intensity.

### Model validation

To account for spatial autocorrelation when evaluating the models and enhance model reliability, we applied a spatial block cross-validation method^64,65^. Specifically, the model calibration area was divided into four separate blocks. Models were fitted by data of three of the blocks and validated using the fourth block, and this process was repeated for all blocks. Then, we estimated the discrimination accuracy of the models using the Boyce index^66^. The continuous Boyce index measures how model predictions differ from a random distribution of the observed presences and is thus a reliable measure of performance for presence-only models^67^. The Boyce index ranges from −1 to 1, with values close to 0 indicating random predictions and close to 1 indicating perfect predictions. Our models performed well for both sets of environmental predictors, achieving median Boyce index values of 0.876 (s.d. = 0.083) and 0.875 (s.d. = 0.086), respectively (Supplementary Fig. 20).

### Model projections

To reduce uncertainties related to each algorithm and enhance the robustness of our predictions, we combined the results of the SDMs into a single ensemble prediction using a weighted average approach with weights proportional to the Boyce evaluations^67^. To guarantee the quality of the ensemble SDMs, we only retained the projections for which the accuracy estimated by the Boyce index was greater than 0.6, indicating good model performance. Subsequently, for each species, we produced ensemble predictions under current and future climatic conditions, employing two future scenarios, two GCMs and two sets of environmental predictors; thus, in total eight ensemble models of future scenarios were calculated for each species. Finally, we transformed the single species ensemble predictions into suitable/non-suitable binary predictions using that single species ensemble model Boyce-maximization threshold, which allowed us to assess predicted plant species richness per site. Given the coarse resolution and incompleteness of native range data, we retained native ranges in the projected species distributions.

### Plant richness calculations and identifying invasion hotspots

The single species binary maps were summed to derive the potential number of alien plant species (that is, potential alien plant richness) in each grid cell under current conditions and future environmental scenarios. To assess uncertainty of the future predictions across models, environmental predictors and GCMs, we calculated the coefficient of variation of predicted values (Supplementary Fig. 10). The coefficient of variation is the ratio of the standard deviation to the mean.

We determined current invasion hotspots as grid cells that were predicted to be suitable for at least 10% (that is, >970 naturalized alien plants) of the modelled species pool under current environmental conditions. To depict potential contractions or expansions of invasion hotspots until the end of the twenty-first century, we defined future invasion hotspots by applying this pre-defined cut-off value established under current conditions (that is, 970 naturalized alien plants). We also calculated the hotspots using different thresholds (that is, 5% and 15% of the modelled species). As expected, the proportion of hotspot areas depended on the threshold. However, the main conclusion remains the same: new hotspots appear in higher latitudes and hotspots disappear in lower ones (Supplementary Fig. 21).

To assess the potential distribution of global naturalized alien plants across biomes, we assigned each grid cell to one of the nine Whittaker biomes^68^ using the Whittaker_biomes function in the R package plotbiomes^69^. Then, we used kernel density smoothers in the ks R package^70^ to calculate the range limits (defined as 95% quantile of the kernel) and range centroid of the global naturalized alien plant ranges. We also calculated the mean number of species per grid cell for the whole study area (that is, the globe) and for each biome separately.

### Addressing uncertainties, biases and accounting for dispersal limitation

We acknowledge that projecting future distributions of species, particularly alien species, is associated with inherent uncertainties and potential biases. By the end of the twenty-first century, it is projected that some regions of the globe will experience novel combinations of climatic conditions, which could affect species distributions in unexpected ways^71^. Geographical barriers, differences in propagule pressure and lag times in spread after introduction will result in a delayed and incomplete filling of the projected suitable environmental space. Conversely, future niche shifts in the new range^72^ may cause spread beyond projected ranges based on current occurrence data. To account for uncertainties associated with future environmental conditions, we used two alternative sets of environmental predictors, two alternative socioeconomic scenarios (SSPs) and two GCMs, implemented in the ensemble prediction. We used these single predictions to calculate the coefficient of variation across models used for the ensemble predictions (Supplementary Fig. 10). To account for differences in the likelihood of range filling due to dispersal limitation, we alternatively calculated plant richness per grid cell and invasion hotspots exclusively based on predicted suitable grid cells located on continents (following the level 1 continents classification of the International Working Group on Taxonomic Databases for Plant Sciences) where a given species is already reported to be naturalized according to GloNAF. Then, we recalculated potential alien plant richness, invasion hotspots and potential distribution of the global naturalized alien plants across biomes. This alternative analysis accounts for the fact that alien plants will only manage to invade environmentally suitable regions once they have overcome dispersal barriers and have been introduced to a continent. The results of this reanalysis showed a lower overall invasion risk compared with the initial non-filtered projections (Supplementary Figs. 11–13). The reduction in predicted invasion risk was more pronounced in the Americas (South and North America) compared with other continents, particularly Europe, where human activities have probably facilitated plant dispersal more intensively and over longer historical periods. Importantly, this sensitivity analysis did not change our main conclusions.

### Reporting summary

Further information on research design is available in the Nature Portfolio Reporting Summary linked to this article.

## Supplementary information

Supplementary Information Supplementary Methods 1, Supplementary Table 1 and Supplementary Figs. 1–21.

Reporting summary

## Data Availability

This study modelled the distribution of the global naturalized alien flora based on publicly available data sources. Single species model results that were used to produce the figures can be accessed at https://bioinvasion-glodi.univie.ac.at/.

## Code availability

The R code of the species distribution models, including data filtering, pseudo-absence selection, individual model fitting, ensemble modelling and future forecasting, can be accessed at https://bioinvasion-glodi.univie.ac.at/.
