# Supplementary material for: The global geography of plant invasion risk under future climate and land-use changes
Source: Nat Ecol Evol. 2026 Mar 27;10(5):952–60. doi: 10.1038/s41559-026-03040-2 (PMC13167448; doi:10.1038/s41559-026-03040-2)
Supplement: Supplementary file 1 — Supplementary Methods 1, Supplementary Table 1 and Supplementary Figs. 1–21. [file 41559_2026_3040_MOESM1_ESM.pdf]

nature ecology & evolution

Supplementary information

https://doi.org/10.1038/s41559-026-03040-2

The global geography of plant invasion risk under future climate and land-use changes

In the format provided by the authors and unedited

Supplementary methods S1: Exploring modeling options to account for uncertainty

We used a random subset of 100 naturalized alien plants to refine our modeling approach before applying it to the full species set, which also substantially reduced the required computational power and time. In particular, we explored two alternative sets of environmental predictors, nine pseudo-absence selection strategies, and five future Global Circulation Models (GCMs) and three Shared Socioeconomic Pathways (SSPs). Each of the two alternative sets of environmental predictors included six variables, namely: (1) annual mean temperature (bio1), temperature annual range (bio7), annual precipitation (bio12), precipitation seasonality [coefficient of variation] (bio15), land use, and soil pH; and (2) temperature seasonality [standard deviation] (bio4), mean temperature of warmest quarter (bio10), precipitation seasonality [coefficient of variation] (bio15), mean monthly precipitation amount of the warmest quarter (bio18), land use, and soil pH). For pseudo-absence selection strategies, we explored nine possible combinations of three different numbers of pseudo-absences drawn; 100, 1,000 and the same number of pseudo-absences as the number of presences available (equal), and three pseudo-absence locations; randomly selected over the calibration area, randomly selected at least two degrees away from any presence point (disk) and randomly selected outside of the suitable area estimated by a surface range envelope (sre) model from the presence records. For GCMs, we explored all five GCMs that are available from CHELSA (i.e., gfdl-esm4, ukesm1-0-ll, mpi-esm1-2-hr, psl-cm6a-lr and mri-esm2-0). For SSPs, we explored a mild scenario SSP1-2.6 (sustainability/taking the green road scenario), mild scenario SSP3-7.0 (regional rivalry – a rocky road) and a severe scenario SSP5-8.5 (fossil-fueled development/taking the highway scenario). For the final model with all species, we kept the two alternative sets of predictor variables as they showed different results, we used pseudo-absence selection strategies with sre and the same number of pseudo-absences as the number of presences available as it resulted in the most accurate models (Fig. S15). For GCMs, we kept

two GCMs that represent relatively low and high predictions of species richness (i.e., ukesm1-0-ll and mpi-esm1-2-hr; Fig. S13,14). Finally, for SSPs, we kept the mild (SSP1) and the severe (SSP5) climate change scenarios (Fig. S13,14)

Table S1: Potential number of naturalized alien plant species in different biomes under current and future environmental scenarios.

**Biome**	**Potential species number (mean)**		
	**Current**	**SSP1**	**SPP5**
Tundra	166.5	314.3	517.1
Boreal forest	529.4	852.4	1008.4
Temperate seasonal forest	1479.2	1549.4	1387.7
Temperate rain forest	998.4	1157.2	1263.7
Tropical rain forest	943.8	988.9	936.3
Tropical seasonal forest/savanna	1207.7	1172.5	1038.8
Subtropical desert	431.4	381.3	331.8
Temperate grassland/desert	627.1	568.2	459.5
Woodland/shrubland	1466.0	1395.9	1075.8

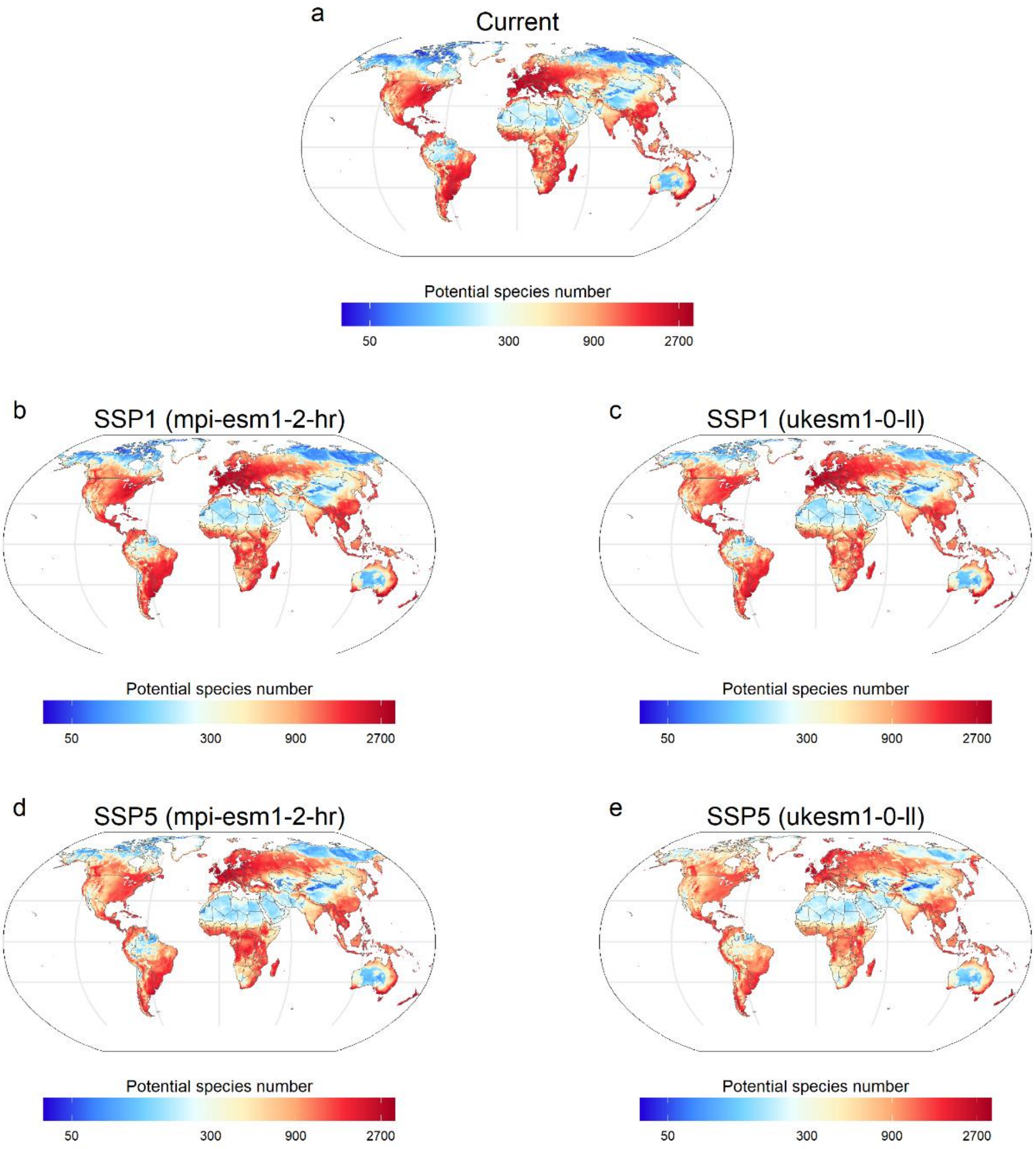

Figure S1: Predicted global patterns of naturalized alien plant richness across 10 × 10 km grid cells. Maps of naturalized alien plants richness under current environmental conditions (a), future environmental change scenarios of projected socioeconomic global changes until the end of the 21st century (b - e). Color coding of species numbers is on log-10 scale and all maps use Robinson projection.

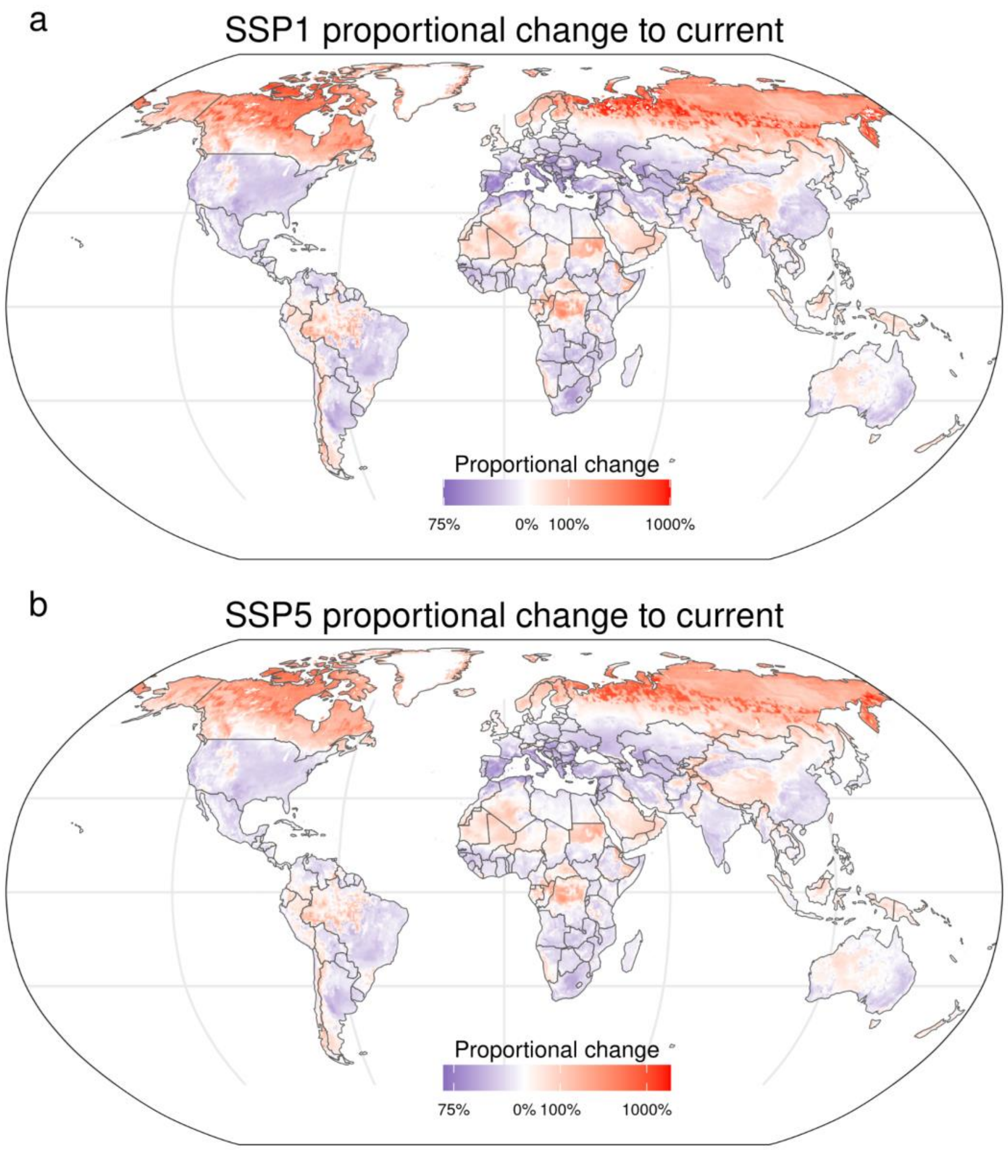

Figure S2: Proportional change of projected alien plant species richness under mild (a) and severe (b) future (2071-2100) climate change compared to current alien plant species richness.

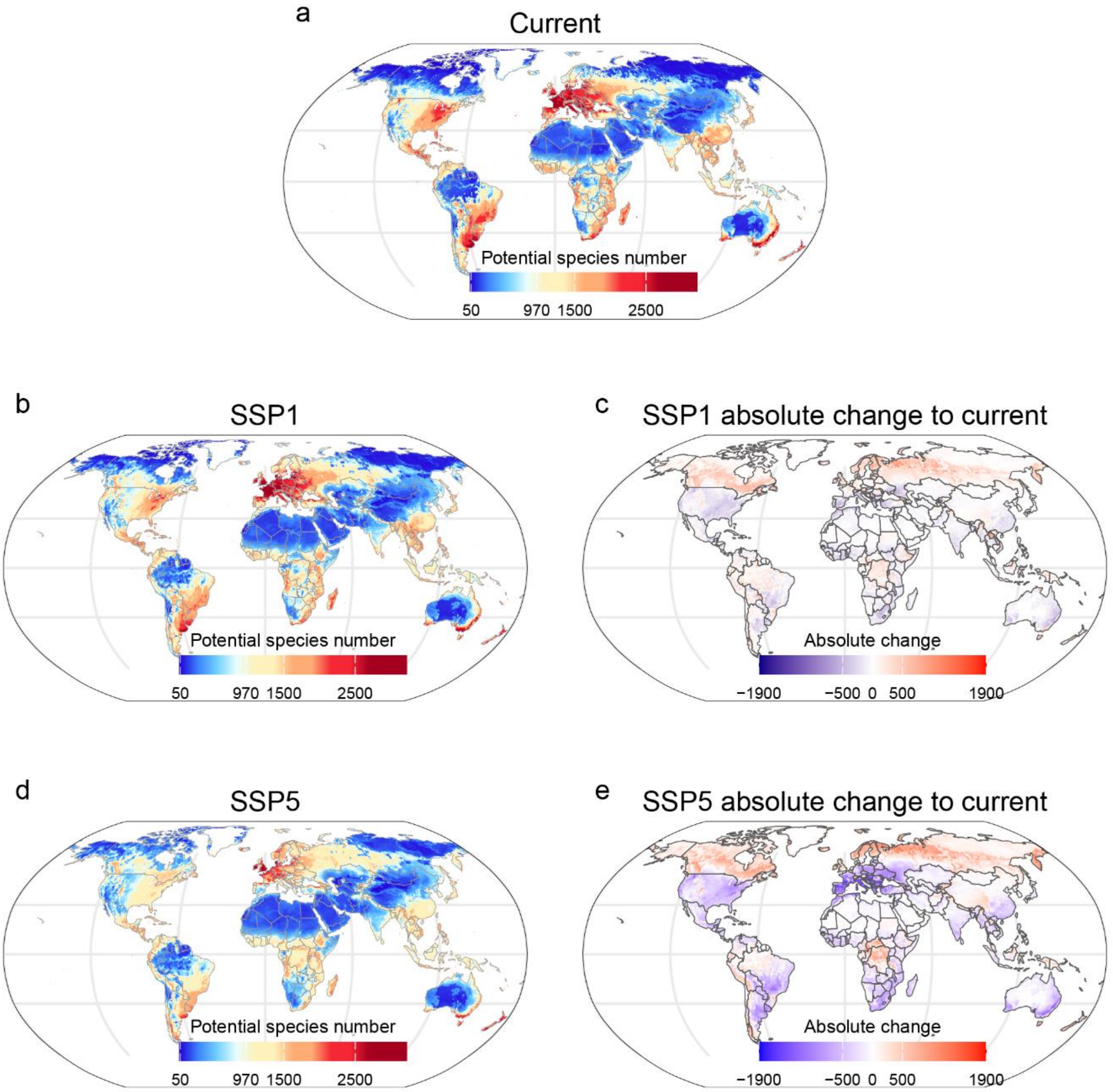

Figure S3: Predicted global patterns of naturalized alien plant richness across 10 × 10 km grid cells. Maps of naturalized alien plants richness under current environmental conditions (a), future mild (b) and severe (d) environmental change scenarios of projected socioeconomic global changes until the end of the 21st century (2071-2100). Predicted richness is based on the ensemble of four models using two different environmental predictor sets and two Global Circulation Models. Absolute change in relation to current richness under future environmental change scenarios: SSP1 (c) and SSP5 (e).

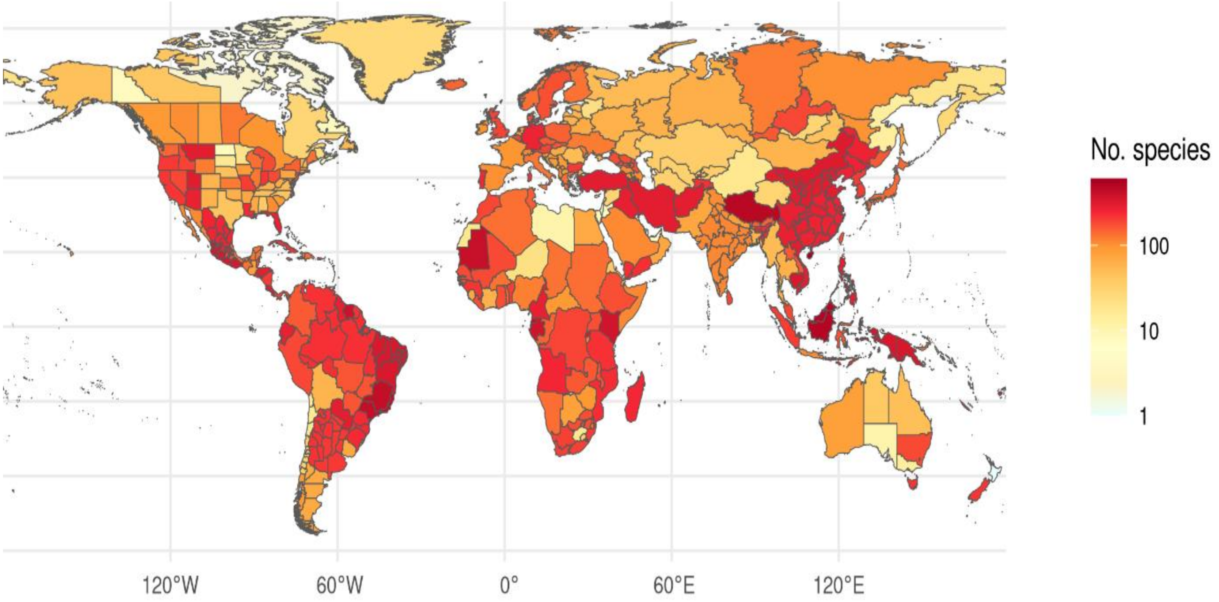

Fig S4: Native distribution of naturalized species that were not modelled due to insufficient data.

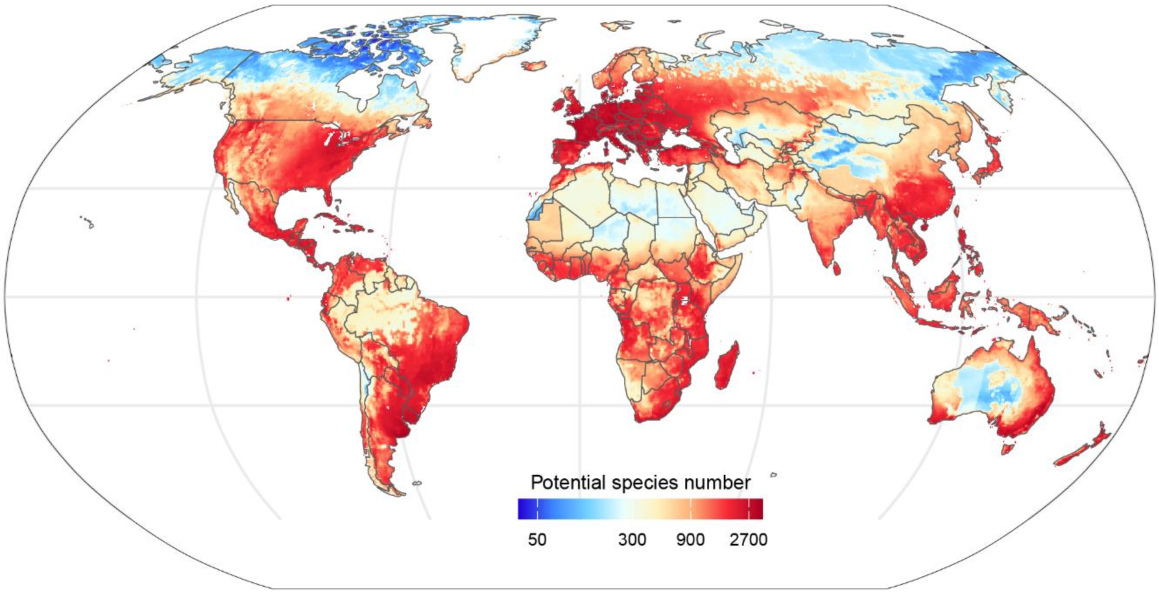

Figure S5: Potential naturalized alien plants richness under current environmental conditions when adding the number of unmodeled species to the potential number of alien plant species in grid cells in the regions where they were reported to be naturalized according to GloNAF.

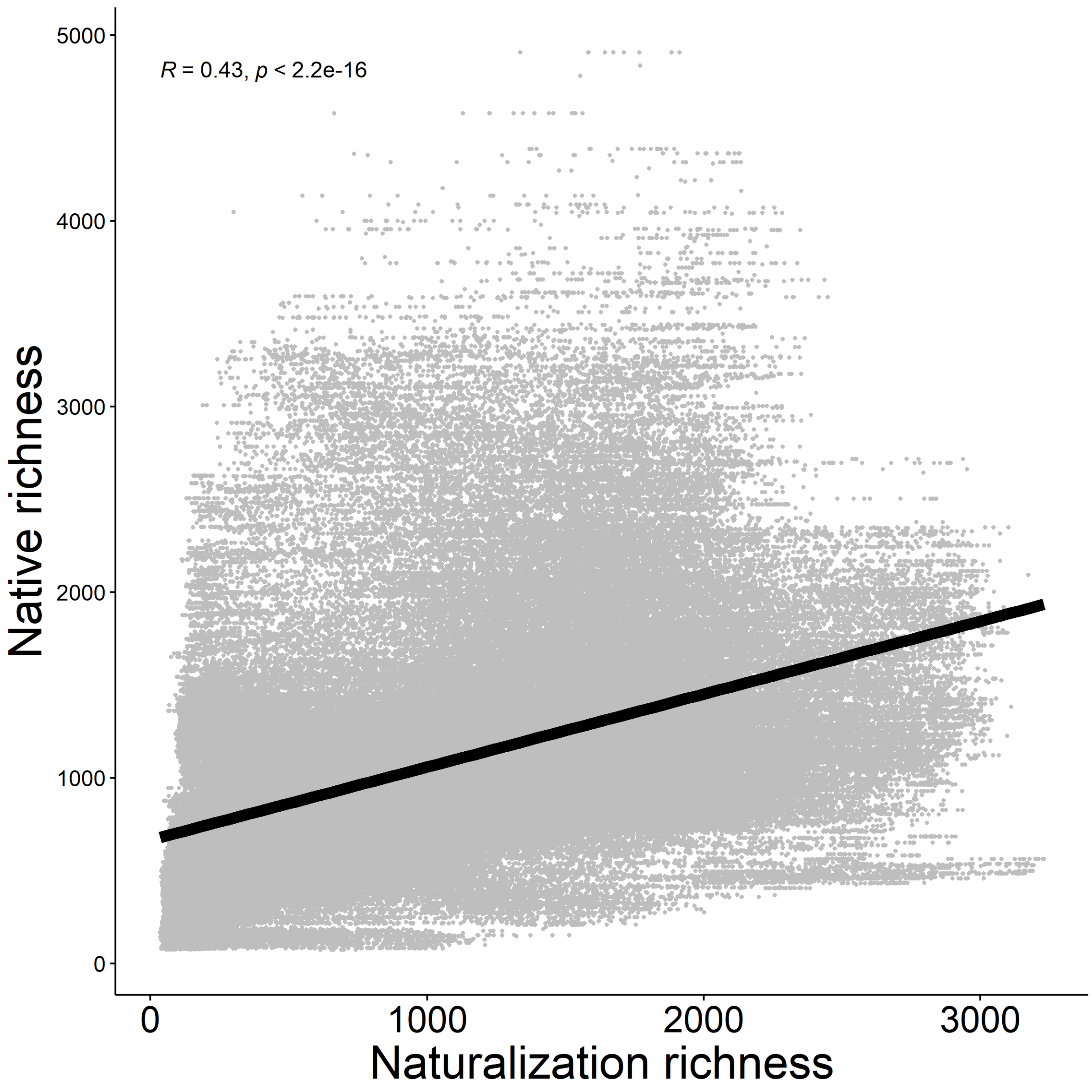

Figure S6: Scatter plot showing the relationship between global native and naturalized alien plant richness. Points represent the number of native and naturalized species in each grid cell. The black solid line represents the linear regression. R represents Pearson's product-moment correlation coefficient.

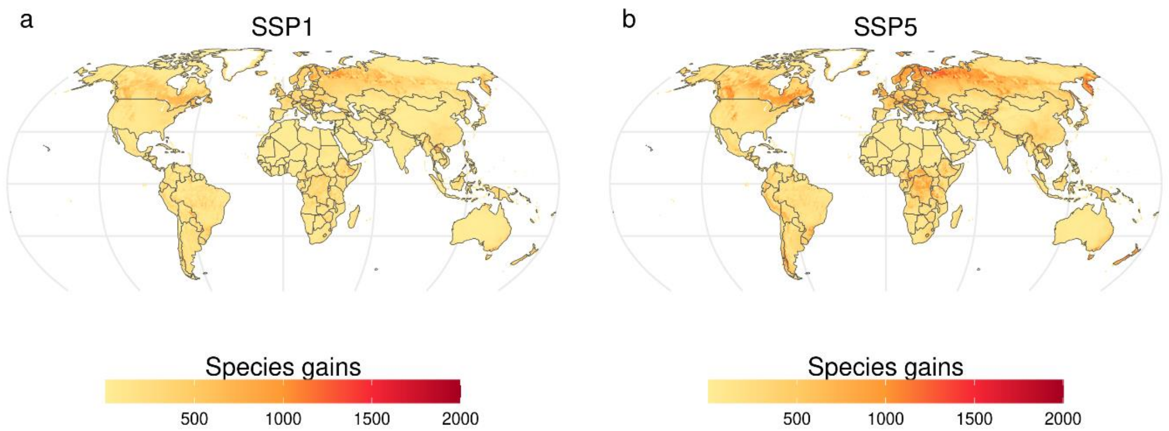

Fig S7: Distribution of naturalized species gains under future environmental scenarios SSP1 (a) and SSP 5 (b).

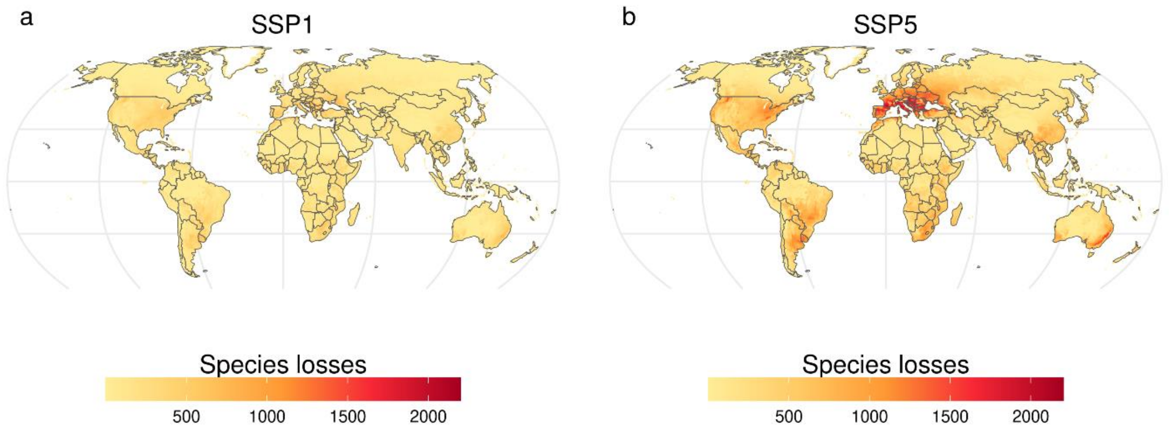

Fig S8: Distribution of naturalized species losses under future environmental scenarios SSP1 (a) and SSP 5 (b).

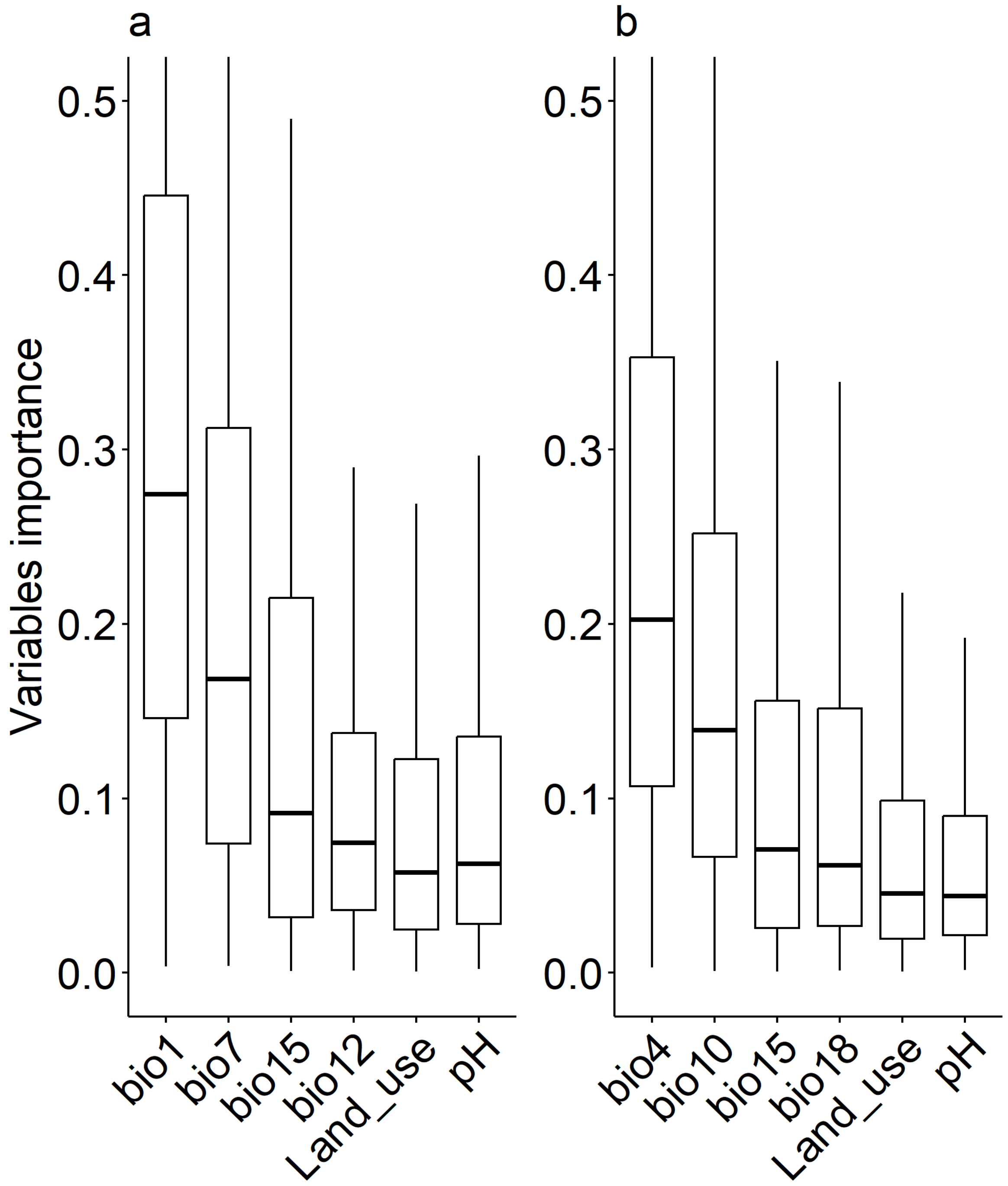

Figure S9: Boxplot of environmental variable importance of the modeled species using the first set of environmental predictors (a) and the second set of environmental predictors (b). The thick horizontal lines in each box indicate the median importance of each environmental variable. The boxes indicate the interquartile range, and the whiskers extend outside the box to 1.5 times the interquartile range. The x axis names indicate the following: annual mean temperature (bio1), temperature annual range (bio7), annual precipitation (bio12), temperature seasonality [standard deviation] (bio4), mean temperature of warmest quarter (bio10), precipitation seasonality [coefficient of variation] (bio15), mean monthly precipitation amount of the warmest quarter (bio15).

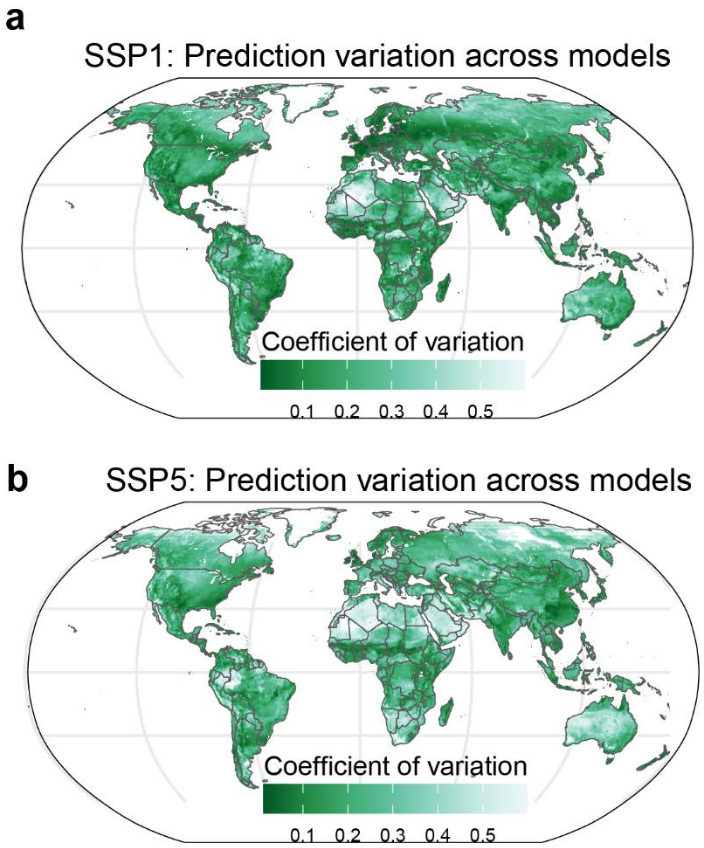

Figure S10: Variation of predictions across models used for the ensemble predictions calculated as coefficient of variation of predicted richness under SSP1 (a) and SSP5 (b).

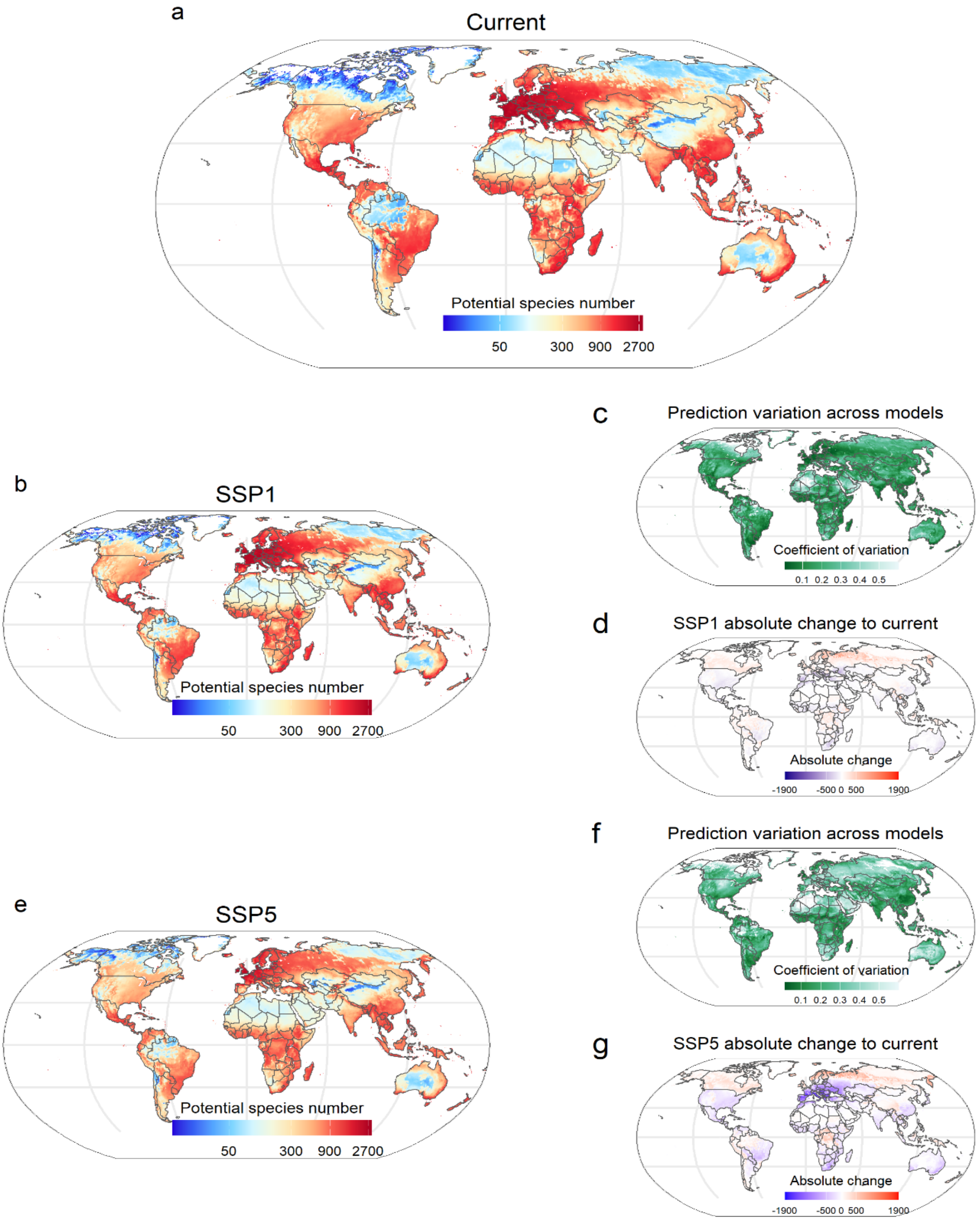

Figure S11: Global patterns of naturalized alien plant richness predicted across 10 × 10 km grid cells filtered to account for dispersal limitation. Maps of naturalized alien plants richness under current (a), future mild (SSP1; b) and severe (SSP5; e) climate change scenarios until the end of the 21st century (2071-2100). Predicted richness is based on the ensemble of four models using two different environmental predictor sets and two GCMs. Variation of predictions across models used for the ensemble predictions is calculated as coefficient of variation of predicted richness under SSP1 (c) and SSP5 (f). Absolute change to current richness under future climate change scenarios: SSP1 (d) and SSP5 (g). In (a, b and e). Color coding of species numbers is on log-10 scale and all maps use Eckert IV projection.

a

b

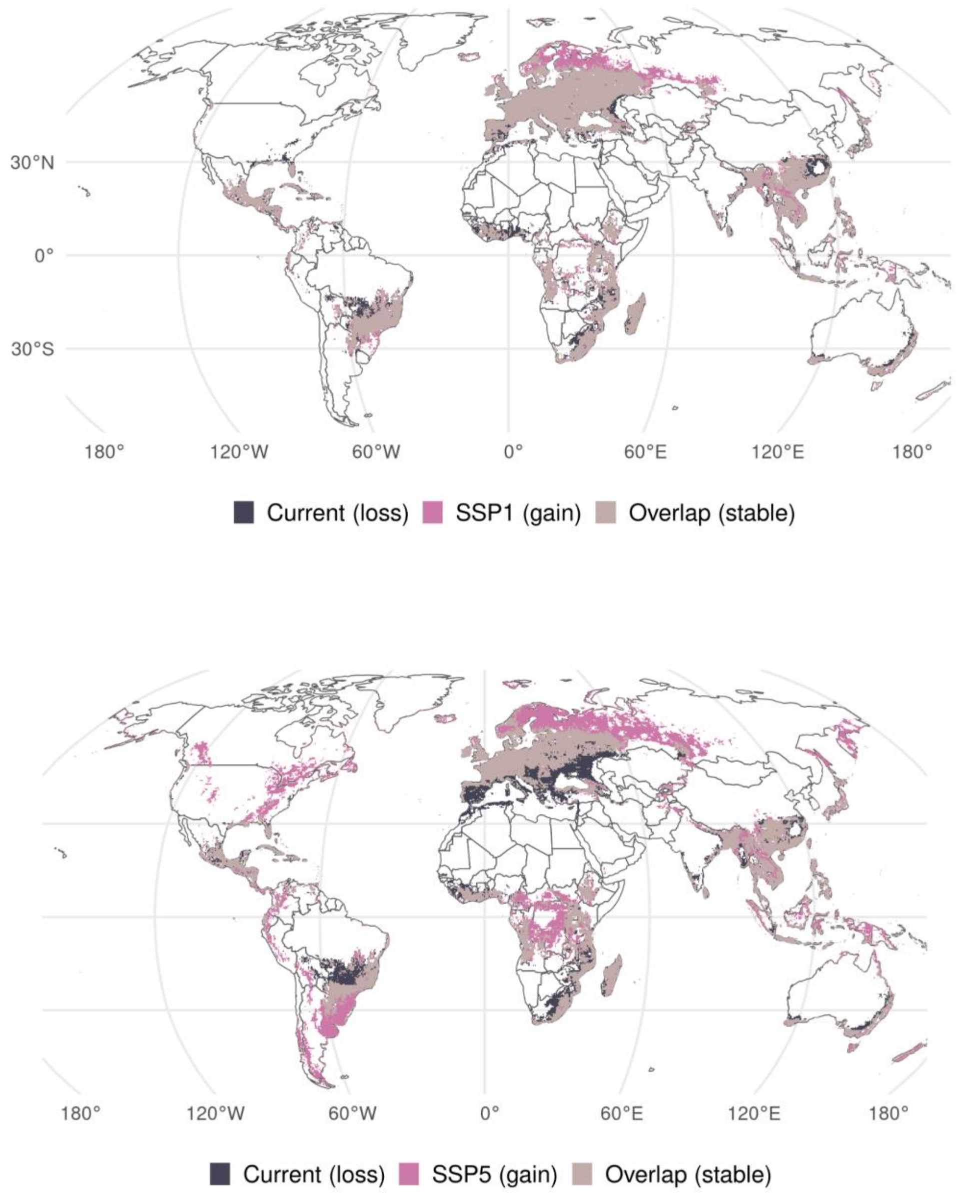

Figure S12: Filtered global hotspots of invasion risk to account for dispersal limitation. Hotspots of invasion risk under current climate and mild (SSP1) (a) and severe (SSP5) (b) climate change scenarios until the end of the 21st century (2071-2100). Invasion hotspots were defined as grid cells that are predicted to be suitable to > 10% (i.e., 970 species) of the modeled species.

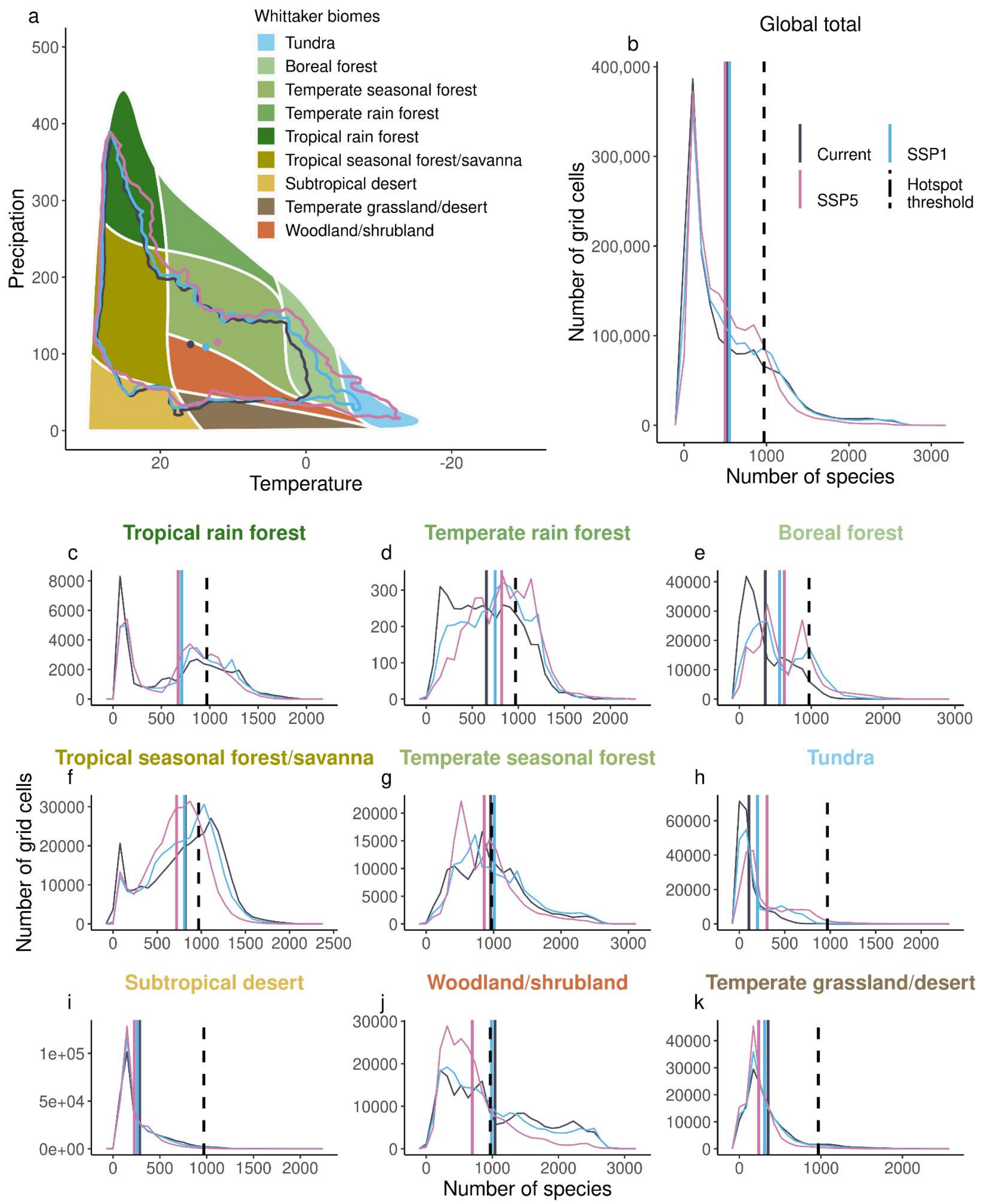

Figure S13: Global current and future potential invasion risk across biomes (Whittaker, 1975) filtered to account for dispersal limitation. Contour lines in (a) show the 95% and centroids of the projected richness of naturalized alien plants under current (dark gray), and mild (SSP1) (light blue) and severe (SSP5) (pink) climate change scenarios until the end of the 21st century (2071-2100). The density plots show the distribution of projected alien plant species richness per grid cell in all biomes (b) and in each of the biomes separately (c-k). Solid vertical lines in each density plot represent the mean species richness under current and future climate change scenarios. Dashed vertical lines represent the hotspot threshold under current environmental conditions (i.e., 970 species).

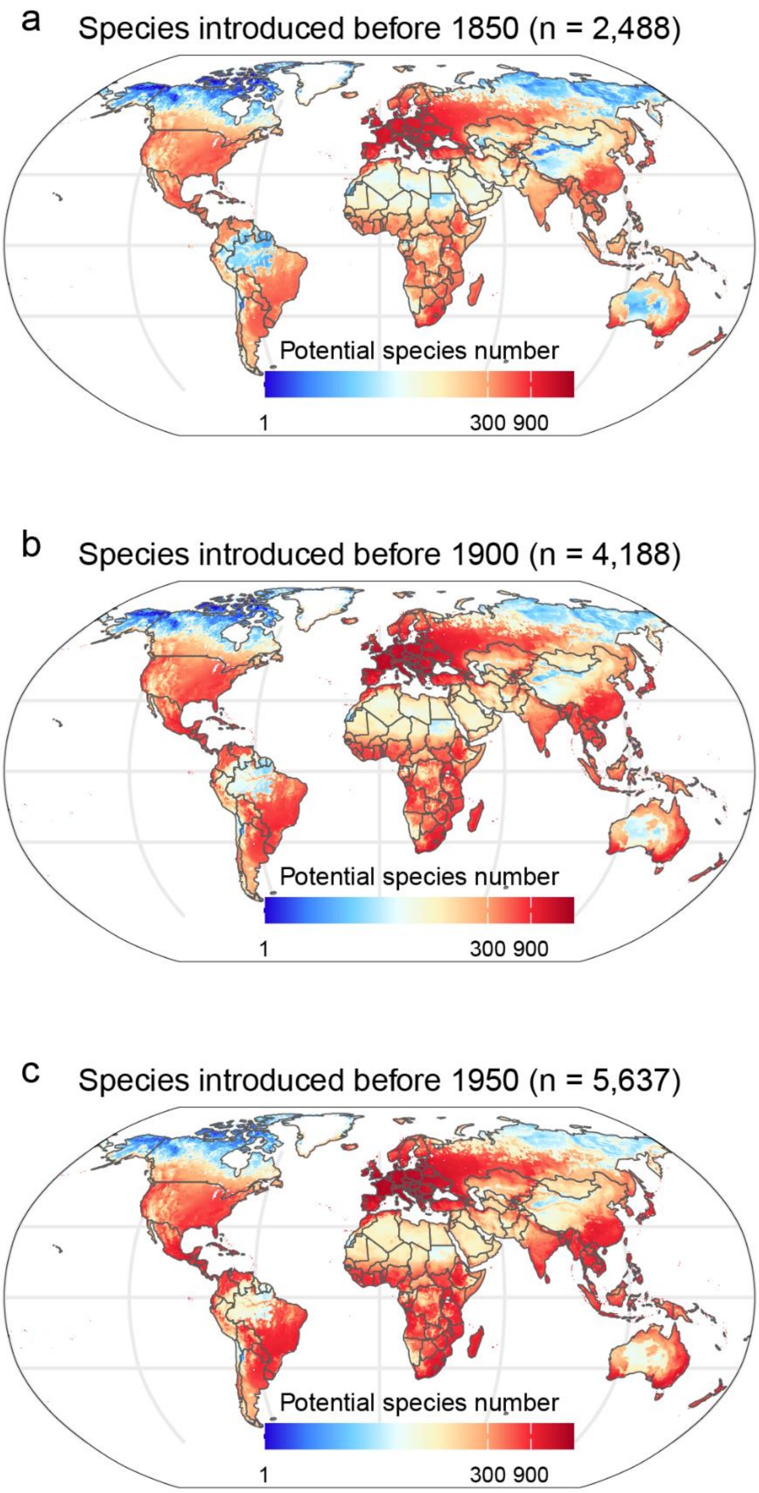

Figure S14: Potential naturalized alien plant species under current environmental conditions using subsets of alien species with first records up to 1850 (a), 1900 (b), and 1950 (c). Color coding of species numbers is on log-10 scale and all maps use Robinson projection.

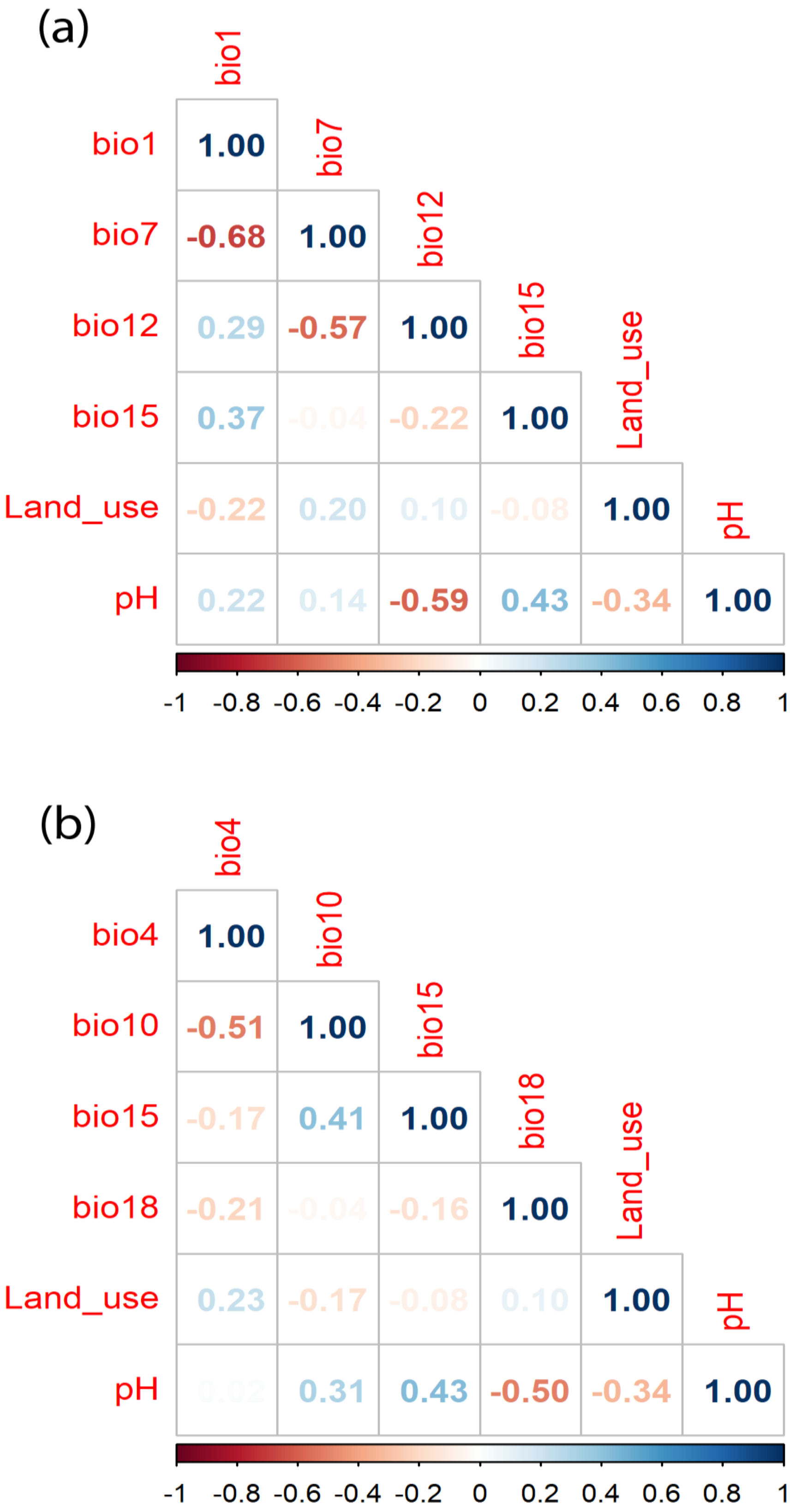

Fig S15: Correlation coefficients between variables used in the first set of environmental predictors (a) and the second set of environmental predictors (b).

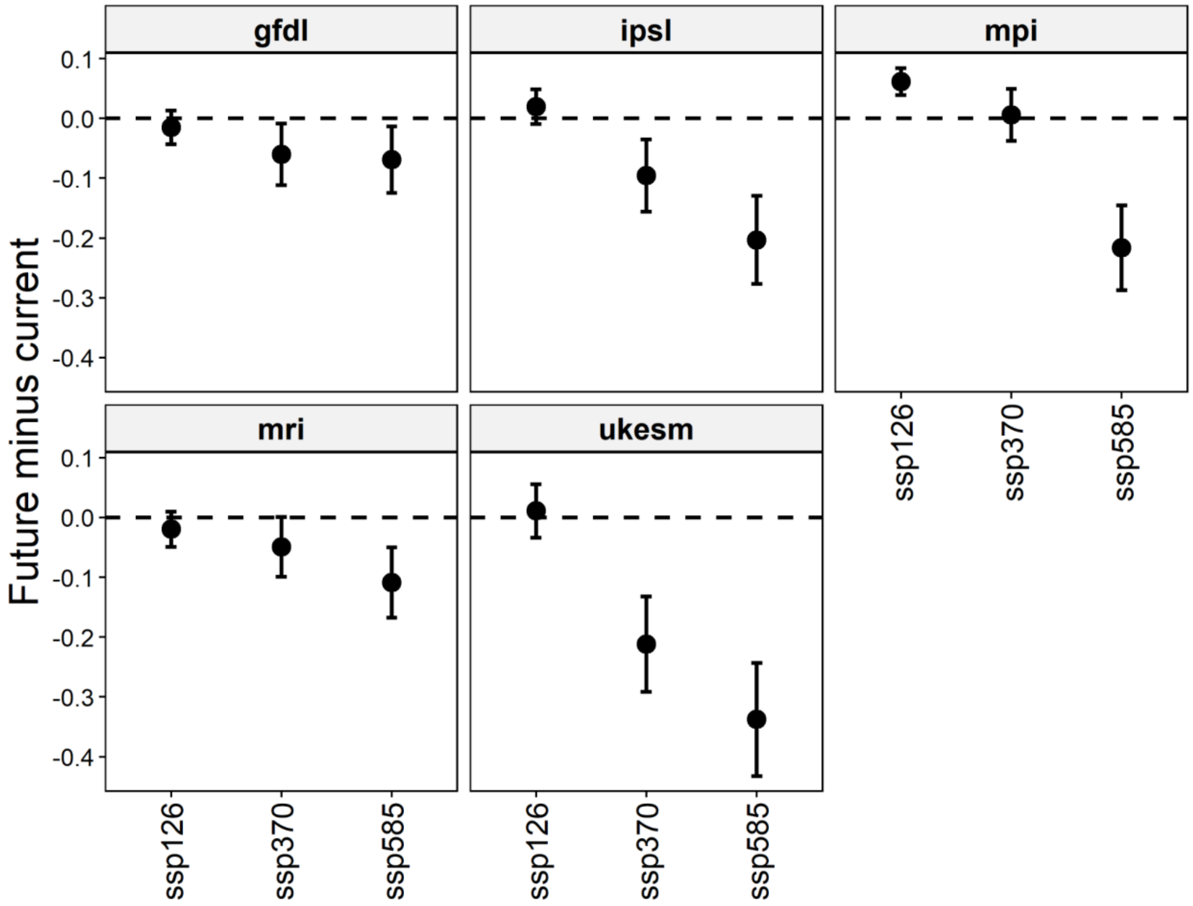

Figure S16: Expected change in future distributions under different shared socioeconomic pathway scenarios (SSP1, SSP3, and SSP5) compared to the current distributions for the 100 subsample species using the first set of environmental predictors. Each panel represents a different GCM; gfdl = gfdl-esm4, ispl = ipsl-cm6a-lr, mpi = mpi-esm1-2-hr, mri = mri-esm2-0, and ukesm = ukesm1-0-ll. Error bars represent the means and 95% confidence intervals of the difference between future and current distributions.

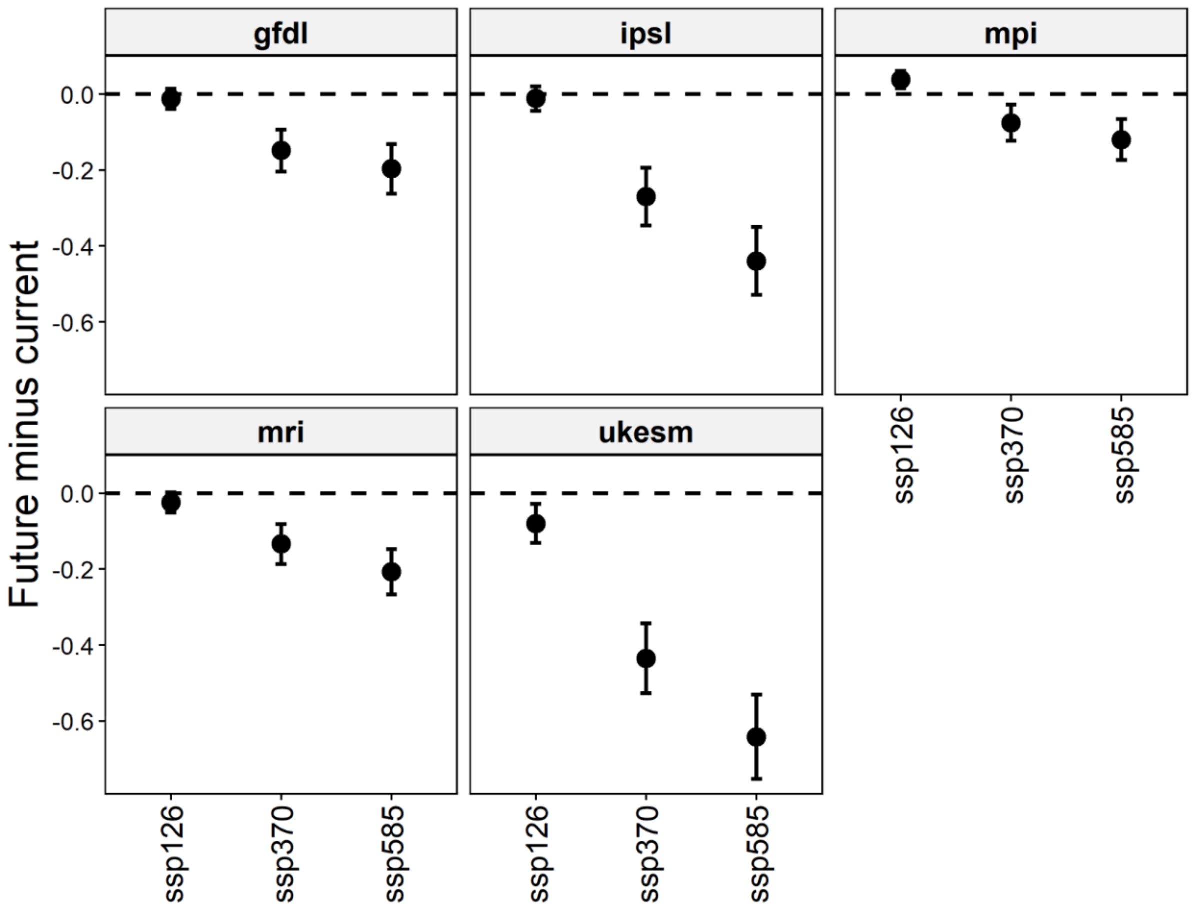

Figure S17: Expected change in future distributions under different shared socioeconomic pathway scenarios (SSP1, SSP3, and SSP5) compared to the current distributions for the 100 subsample species using the second set of environmental predictors. Each panel represents a different GCM; gfdl = gfdl-esm4, ispl = ipsl-cm6a-lr, mpi = mpi-esm1-2-hr, mri = mri-esm2-0, and ukesm = ukesm1-0-ll. Error bars represent the means and 95% confidence intervals of the difference between future and current distributions.

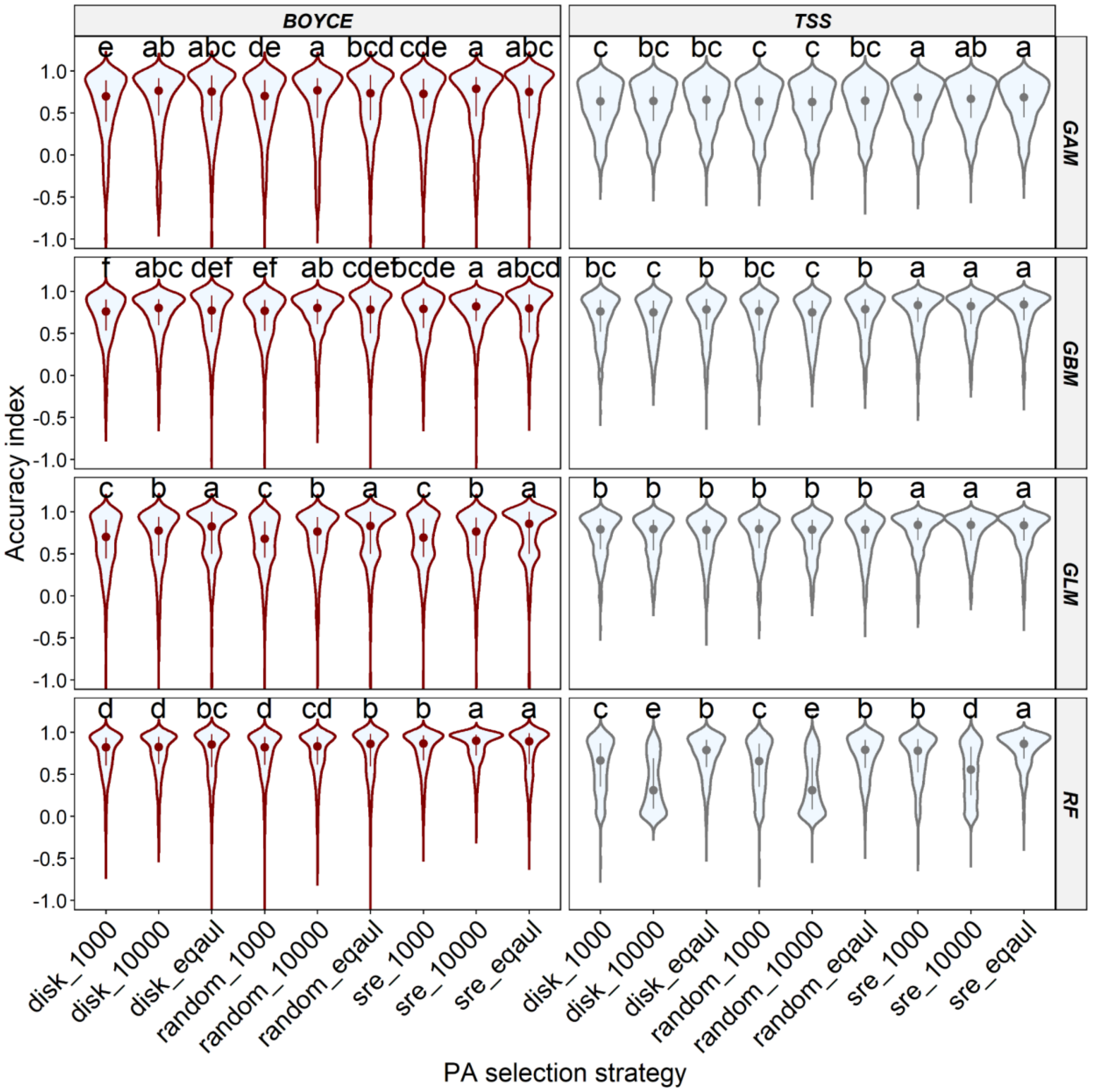

Figure S18: Evaluations of the different pseudo-absence (PA) selection strategies using Boyce (red) and TSS (grey) indices using the first set of environmental predictors. Violins represent the distribution of the Boyce and TSS values. Dots and vertical lines inside each violin represent medians and 95% confidence intervals. Different letters indicate statistically significant differences ($P \leq 0.05$) between the medians of the different strategies. We test for differences between medians using the 'Median.test' function of the 'agricolae' R package.

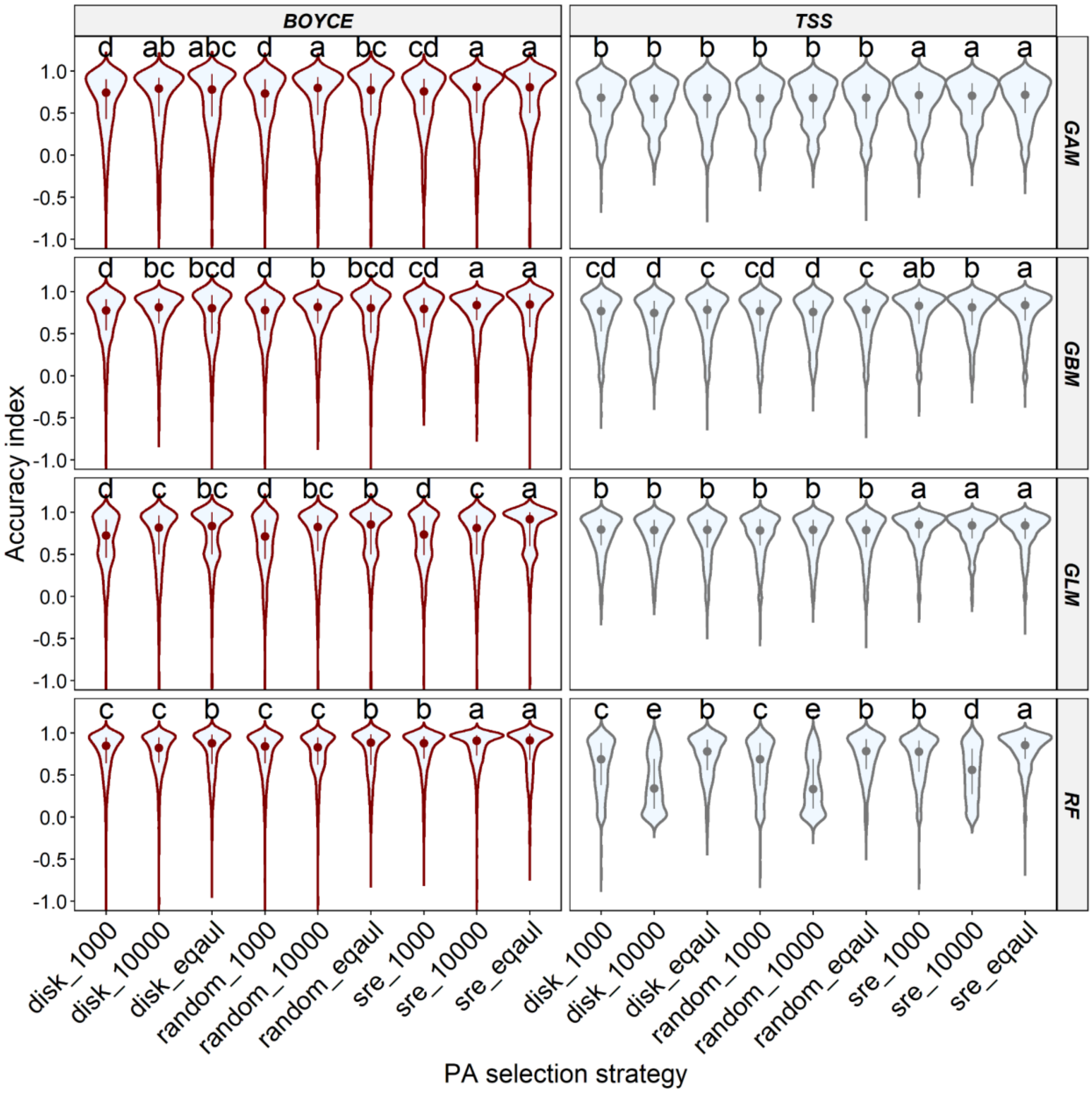

Figure S19: Evaluations of the different pseudo-absence (PA) selection strategies using Boyce (red) and TSS (grey) indices using the second set of environmental predictors. Violins represent the distribution of the Boyce and TSS values. Dots and vertical lines inside each violin represent medians and 95% confidence intervals. Different letters indicate statistically significant differences ($P \leq 0.05$) between the medians of the different strategies. We test for differences between medians using the ‘Median.test’ function of the ‘agricolae’ R package.

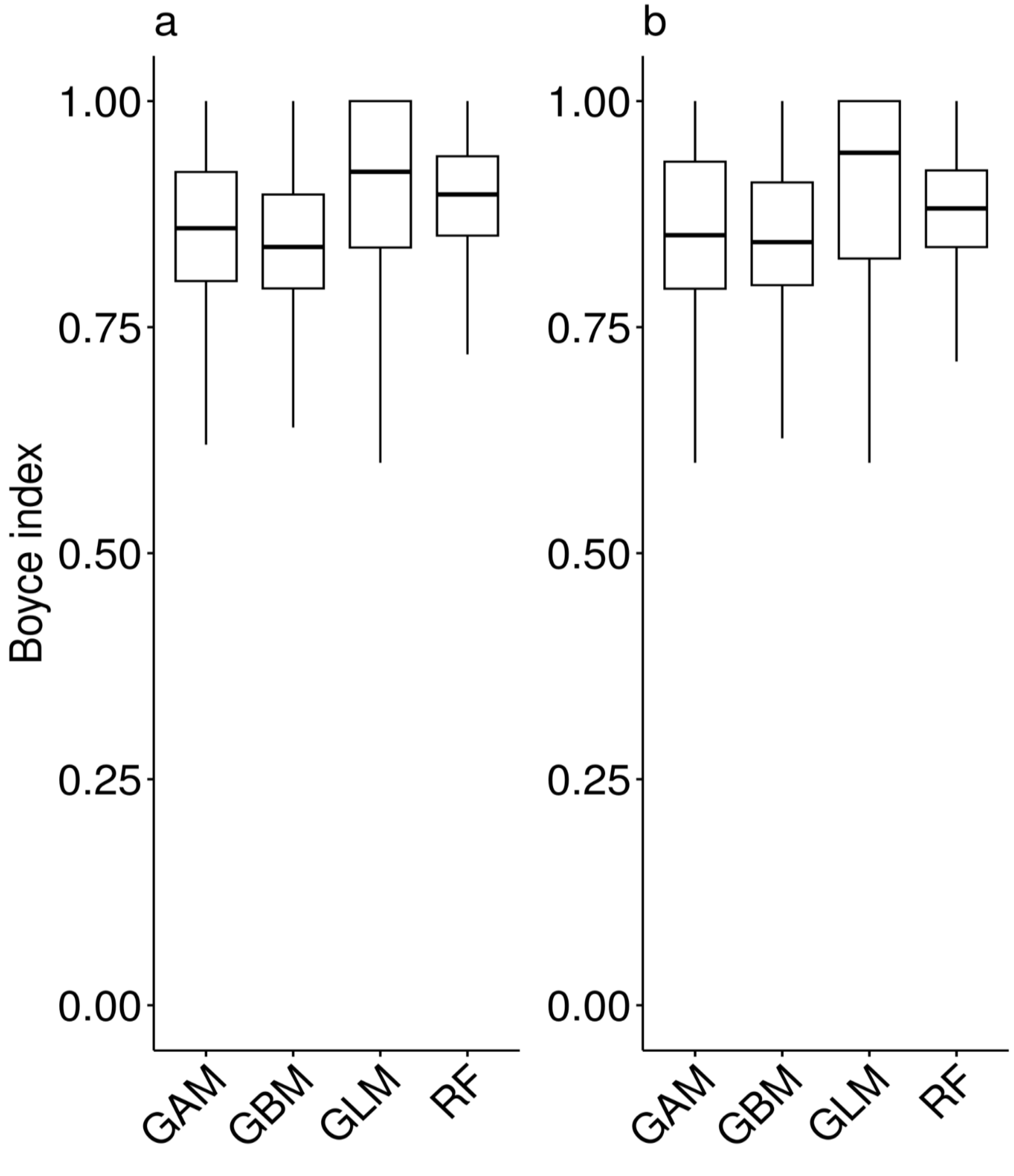

Figure S20: Boxplot of model algorithm accuracy using the first set of environmental predictors (a) and the second set of environmental predictors (b). The thick horizontal lines in each box indicate the median importance of each environmental variable. The boxes indicate the interquartile range, and the whiskers extend outside the box to 1.5 times the interquartile range. The overall median equals 0.876 and 0.875 in a and b, respectively.

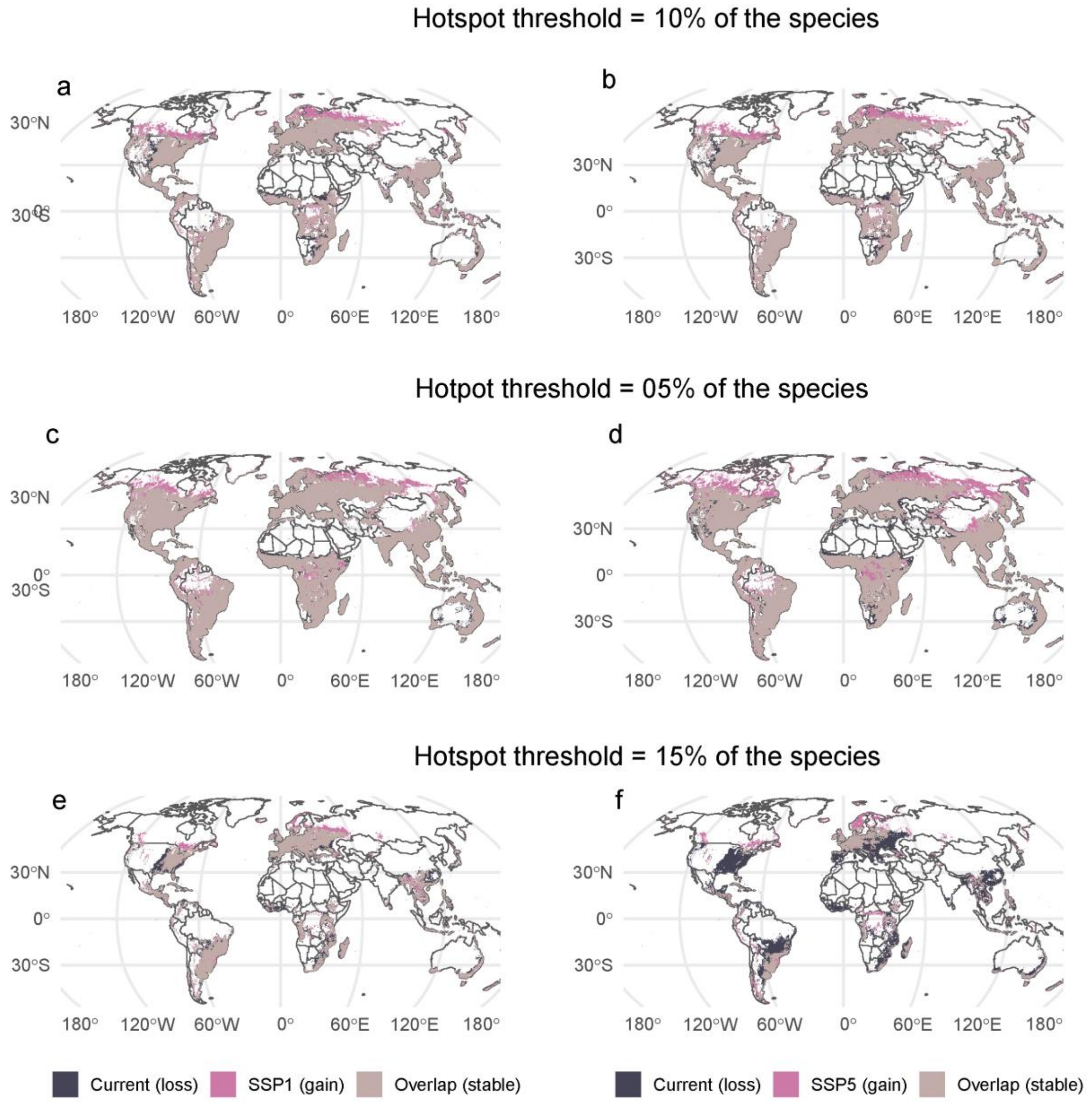

Figure S21: Global hotspots of invasion risk. Change in proportion and spatial distribution of invasion hotspots under current environmental conditions and mild (a, c, and e) and severe (b, d,and f) environmental change scenarios until the end of the 21st century (2071-2100). Invasion hotspots were defined as grid cells that are predicted to be suitable to >5%, > 10%, and >15% of the modeled species (i.e., 485, 970, and 1455, respectively).
