# Supplementary material for: The global geography of plant invasion risk under future climate and land-use changes
Source: Nat Ecol Evol. 2026 Mar 27;10(5):952–60. doi: 10.1038/s41559-026-03040-2 (PMC13167448; doi:10.1038/s41559-026-03040-2)
Supplement: Supplementary file 2 — Reporting summary [file 41559_2026_3040_MOESM2_ESM.pdf]

nature portfolio

Corresponding author(s): Ali Omer

Last updated by author(s): Feb 23, 2026

Reporting Summary

Nature Portfolio wishes to improve the reproducibility of the work that we publish. This form provides structure for consistency and transparency in reporting. For further information on Nature Portfolio policies, see our Editorial Policies and the Editorial Policy Checklist.

Statistics

For all statistical analyses, confirm that the following items are present in the figure legend, table legend, main text, or Methods section.

n/a	Confirmed	
☐	☒	The exact sample size (*n*) for each experimental group/condition, given as a discrete number and unit of measurement
☒	☐	A statement on whether measurements were taken from distinct samples or whether the same sample was measured repeatedly
☐	☒	The statistical test(s) used AND whether they are one- or two-sided<br>*Only common tests should be described solely by name; describe more complex techniques in the Methods section.*
☐	☒	A description of all covariates tested
☒	☐	A description of any assumptions or corrections, such as tests of normality and adjustment for multiple comparisons
☐	☒	A full description of the statistical parameters including central tendency (e.g. means) or other basic estimates (e.g. regression coefficient) AND variation (e.g. standard deviation) or associated estimates of uncertainty (e.g. confidence intervals)
☐	☒	For null hypothesis testing, the test statistic (e.g. *F*, *t*, *r*) with confidence intervals, effect sizes, degrees of freedom and *P* value noted<br>*Give P values as exact values whenever suitable.*
☒	☐	For Bayesian analysis, information on the choice of priors and Markov chain Monte Carlo settings
☒	☐	For hierarchical and complex designs, identification of the appropriate level for tests and full reporting of outcomes
☐	☒	Estimates of effect sizes (e.g. Cohen's *d*, Pearson's *r*), indicating how they were calculated

Our web collection on statistics for biologists contains articles on many of the points above.

Software and code

Policy information about availability of computer code

Data collection: Data collected with use of no software.

Data analysis: The main data processing and analysis were done using R (version 4.4.0.) and Python (version 3.13). We used R packages 'biomod2' version 4.2-5-2 and flexsdm version 1.3.9 to run the species distribution models (SDMs). We used R package CoordinateCleaner version 3.0.1 to clean species distribution data. R Packages used to for data formatting, analyses, and plotting: tidyverse version 2.0.0, corrplot version 0.95, ggpubr version 0.6.1, terra version 1.7.65, ggplot2 version 4.0.0, plotbiomes version 0.0.0.9001, and ks version 1.15.1.

For manuscripts utilizing custom algorithms or software that are central to the research but not yet described in published literature, software must be made available to editors and reviewers. We strongly encourage code deposition in a community repository (e.g. GitHub). See the Nature Portfolio guidelines for submitting code & software for further information.

Data

Policy information about availability of data

All manuscripts must include a data availability statement. This statement should provide the following information, where applicable:

- Accession codes, unique identifiers, or web links for publicly available datasets
- A description of any restrictions on data availability
- For clinical datasets or third party data, please ensure that the statement adheres to our policy

Provide your data availability statement here.

Research involving human participants, their data, or biological material

Policy information about studies with human participants or human data. See also policy information about sex, gender (identity/presentation), and sexual orientation and race, ethnicity and racism.

Reporting on sex and gender	n/a
Reporting on race, ethnicity, or other socially relevant groupings	n/a
Population characteristics	n/a
Recruitment	n/a
Ethics oversight	n/a

Note that full information on the approval of the study protocol must also be provided in the manuscript.

Field-specific reporting

Please select the one below that is the best fit for your research. If you are not sure, read the appropriate sections before making your selection.

☐ Life sciences ☐ Behavioural & social sciences ☒ Ecological, evolutionary & environmental sciences

For a reference copy of the document with all sections, see nature.com/documents/nr-reporting-summary-flat.pdf

Ecological, evolutionary & environmental sciences study design

All studies must disclose on these points even when the disclosure is negative.

Study description	In this study, we used Species Distribution Models (SDMs) to project the current and future potential distributions of naturalized alien plant species, until the end of the 21st century. Using the single species projections, we mapped potential hotspots of plant invasions, predicted shifts in these hotspots across biogeographic biomes, and assessed the potential turnover within regional naturalized plant pools. Our results reveal that the global average number of naturalized alien plants will change only moderately under future environmental change scenarios. However, this apparent stasis of global averages masks considerable spatial and compositional rearrangement of naturalized alien plant richness and invasion hotspots.
Research sample	The study included 9,701 species, representing 70.2% of the global naturalized alien flora.
Sampling strategy	We based the selection of the global naturalized alien plant pool on the most comprehensive data source, i.e., GloNAF (Global Naturalized Alien Flora) database. We used GloNAF V.2.16, which contains 16,429 taxa (i.e., species, subspecies and varieties) that are naturalized in at least one region globally.
Data collection	The GloNAF dataset was developed by the authors. Other datasets used in this study were downloaded by the corresponding author.
Timing and spatial scale	GloNAF dataset was accessed on June 2024. Soil, climate and land use datasets were accessed on November 2023. Geo-referenced occurrences data was accessed on February 2024. All datasets used in this study are at the global scale.
Data exclusions	Infraspecific taxa (e.g., subspecies, varieties) were removed from the species list to to reduce complexity. To ensure accurate and robust SDMs predictions, we only kept species with at least 30 occurrence records.
Reproducibility	As our study mainly analyzed existing datasets, we did not test for reproducibility. However, we will provide a link for all the processed data and code that could be used to reproduce the main results of the study.
Randomization	We mainly analyzed existing datasets, so we did not allocate samples to groups.
Blinding	We mainly analyzed existing datasets, so blinding was not relevant to this study.

Did the study involve field work? ☐ Yes ☒ No

Reporting for specific materials, systems and methods

We require information from authors about some types of materials, experimental systems and methods used in many studies. Here, indicate whether each material, system or method listed is relevant to your study. If you are not sure if a list item applies to your research, read the appropriate section before selecting a response.

Materials & experimental systems

n/a	Involved in the study
☒	☐ Antibodies
☒	☐ Eukaryotic cell lines
☒	☐ Palaeontology and archaeology
☒	☐ Animals and other organisms
☒	☐ Clinical data
☒	☐ Dual use research of concern
☒	☐ Plants

Methods

n/a	Involved in the study
☒	☐ ChIP-seq
☒	☐ Flow cytometry
☒	☐ MRI-based neuroimaging

Plants

Seed stocks	n/a
Novel plant genotypes	n/a
Authentication	n/a
